# Trends and projections of colorectal carcinoma mortality in U.S. adults, 1999–2050: A CDC WONDER-based ecological time-series analysis

**DOI:** 10.1097/MD.0000000000051146

**Published:** 2026-10-16

**Authors:** Kanza Atif, Eshal Atif, Vishan Das, Sadia Qazi, Mazhar Ali, Abdal Ahmad, Fnu Urooba, Mohib Ali Hassan, Muhammad Mukhlis, Muhammad Atif Mazhar, Akef Obeidat

**Affiliations:** a College of Medicine, Alfaisal University, Riyadh, Saudi Arabia; b Liaquat University of Medical & Health Sciences, Jamshoro, Sindh, Pakistan; c Department of Anatomy, College of Medicine, Alfaisal University, Riyadh, Saudi Arabia; d Khyber Medical College, Peshawar, Pakistan; e Department of Medicine, Peshawar Medical College, Riphah International University (Peshawar Campus), Peshawar, Pakistan; f CMH Institute of Medical Sciences, Multan, Pakistan; g Rawal Institute of Health Sciences, Islamabad, Pakistan; h Ayub Medical College, Abbottabad, Pakistan.

**Keywords:** age-adjusted mortality rate, CDC WONDER, colorectal cancer mortality, colorectal carcinoma, early-onset colorectal cancer, health disparities, joinpoint regression, mortality projection

## Abstract

Colorectal carcinoma (CRC) remains a major cause of cancer mortality in the United States (U.S.); however, contemporary long-term projections by demographic and geographic strata are limited. This study examined national colorectal cancer mortality trends from 1999 to 2025 and projected age-adjusted mortality rates (AAMRs) through 2050. We conducted an ecological time-series analysis of U.S. adults aged ≥ 25 years using Centers for Disease Control and Prevention Wide-ranging Online Data for Epidemiologic Research Multiple Cause of Death data. CRC-related deaths were identified by any mention of International Classification of Diseases, Tenth Revision codes C18–C20 on the death certificate. AAMRs per 100,000 population were calculated overall and stratified by sex, age group, race and ethnicity, urbanization, census region, state, and place of death. Joinpoint regression was used to estimate the annual percent change and average annual percent change. A demographically optimized ensemble forecasting model was used to project AAMRs from 2026 to 2050. From 1999 to 2025, 1,674,393 CRC-related deaths were recorded. Overall, the AAMR declined from 38.11 in 1999 to 23.14 in 2025 (average annual percent change, −1.99%; 95% confidence interval, −2.13 to −1.85; *P* < .001), but recent rates have stabilized. The mean AAMR was higher among men than women (34.13 vs 23.43) and higher in nonmetropolitan than metropolitan areas (32.43 vs 28.49). Non-Hispanic Black adults had the highest racial and ethnic burdens (37.45). Adults aged 25 to 44 years showed a significant increase in mortality (annual percent change, 1.13%; 95% confidence interval, 0.93 to 1.33; *P* < .001). Most deaths occurred at home (41.15% of deaths). The overall AAMR was projected to continue declining slowly, from 23.02 in 2026 to 21.56 in 2050, at a slower pace than the 1999 to 2016 decline. U.S. CRC mortality has declined substantially and is projected to continue declining slowly through 2050, rather than resume its earlier faster decline, with persistent sex, racial, rural, age, and geographic disparities. Targeted strategies are needed to improve screening completion, diagnostic follow-up, rural access, equity, and early-onset colorectal cancer detection.

## 1. Introduction

Colorectal carcinoma (CRC) is one of the most common malignancies and remains a major contributor to cancer-related mortality in the United States (U.S.). It is the third most commonly diagnosed cancer and the third leading cause of cancer-related death, with an estimated 151,030 new cases and 52,580 deaths reported in 2022.^[1]^ Although the overall incidence and mortality of colorectal cancer have declined over recent decades, largely because of screening uptake, earlier detection, removal of premalignant lesions, and improvements in treatment, the disease continues to impose a substantial clinical, public health, and economic burden.^[1,2]^

Aggregate improvements in CRC outcomes conceal important inequalities across population groups. Prior studies have documented important differences in the colorectal cancer burden by age, race and ethnicity, subsite, and stage.^[3,4]^ Simultaneously, the rising incidence of early-onset colorectal cancer among adults younger than 50 years has raised new concerns regarding future mortality patterns, particularly because younger patients may be diagnosed at later stages and outside traditional screening pathways.^[3]^ These shifts indicate that historical declines in mortality may not continue uniformly across all demographic and geographic categories.

Existing research has extensively described the incidence, mortality, screening, and survival patterns of colorectal cancer in the U.S.^[2,4]^ However, much of the available evidence is based on historical surveillance periods and does not provide long-horizon projections extending to 2050 using recent national mortality data. Global projection studies have estimated the future burden of colorectal cancer; however, they often aggregate countries or regions and may not fully resolve the subgroup-specific patterns most relevant to U.S. public health planning.^[5]^ Therefore, a contemporary national analysis that combines long-term observed mortality trends with subgroup-specific forecasts is needed to identify where the future CRC mortality burden is likely to concentrate.

The rationale for this study is based on the need to anticipate future CRC mortality so that health systems and public health agencies can plan screening, prevention, diagnosis, treatment, and survivorship resources more effectively. Changes in obesity, dietary patterns, physical inactivity, smoking, alcohol use, and screening practices may alter future mortality trajectories.^[6,7]^ Forecasting mortality through 2050 can help identify whether current declines are expected to continue, stabilize, or reverse and whether specific groups require intensified prevention and early detection strategies.

We analyzed national age-adjusted CRC mortality among U.S. adults aged ≥ 25 years from 1999 to 2025 using the Centers for Disease Control and Prevention Wide-ranging Online Data for Epidemiologic Research (CDC WONDER) Multiple Cause of Death database. We characterized temporal trends using joinpoint regression and projected mortality rates through 2050 using a demographically optimized ensemble-forecasting model. Our objective was to quantify overall and subgroup-specific CRC mortality trends by sex, age group, race and ethnicity, urbanization, census region, and state, and to identify populations and geographic areas that may carry a disproportionate future mortality burden.

## 2. Methods

### 2.1. Study design and data source

We conducted an ecological time-series analysis using the CDC WONDER Multiple Cause of Death public-use database. CDC WONDER mortality data are derived from death certificates filed in all 50 states and the District of Columbia and include information on the underlying cause of death, contributing causes of death, demographic characteristics, place of death, and geographic identifiers.^[8]^

CRC-related deaths were identified using the International Classification of Diseases, Tenth Revision (ICD-10) codes for malignant neoplasms of the colon, rectosigmoid junction, and rectum. The included ICD-10 codes were C18 for malignant neoplasm of the colon, C19 for malignant neoplasm of the rectosigmoid junction, and C20 for malignant neoplasm of the rectum.^[9]^ Deaths coded as malignant neoplasms of the anus and anal canal, including C21, were excluded because anal cancer is anatomically and epidemiologically distinct from standard CRC.^[9]^

The analytic window was from January 1, 1999, to December 31, 2025. Deaths were included when CRC was listed anywhere on the death certificate as either the underlying or contributing cause. Because a multiple-cause (any-mention) definition was used, the counts and rates were higher than those based on the underlying cause alone. Mortality counts for 1999 to 2024 were obtained from the final Multiple Cause of Death files, and 2025 counts were obtained from the CDC WONDER Provisional Mortality file; the provisional 2025 data are subject to later revision and may undercount deaths. Data were extracted from the CDC WONDER on March 16, 2026. Because this study used publicly available deidentified mortality data, institutional review board approval and informed consent were not required. The reporting followed the Strengthening the Reporting of Observational Studies in Epidemiology guidelines.^[10]^

### 2.2. Population and stratification

The analysis included all CRC-related deaths among U.S. adults aged ≥ 25 years during the study period. Age was stratified into 3 groups: 25 to 44, 45 to 64, and 65 to 85 + years. Sex was classified as male or female.

Race and ethnicity were recoded into 5 mutually exclusive CDC WONDER categories: Hispanic or Latino, non-Hispanic (NH) White, NH Black or African American, NH American Indian or Alaska Native, and NH Asian or Pacific Islander. The place of death was categorized as the decedent’s home, medical facility inpatient, medical facility outpatient or emergency room, medical facility dead on arrival, medical facility status unknown, hospice facility, nursing home or long-term care facility, other, and unknown.

County-level urbanization was classified using the National Center for Health Statistics Urban–Rural Classification Scheme and was collapsed into metropolitan and nonmetropolitan categories.^[11]^ Census regions were defined according to the standard U.S. Census Bureau classification as Northeast, Midwest, South, and West. State-level analyses were conducted separately for 1999 to 2020 and 2021 to 2025 to describe changes in the state-level mortality burden across earlier and more recent periods.

### 2.3. Outcome measures

The primary outcome was the annual age-adjusted mortality rate (AAMR) per 100,000 population. AAMRs were standardized to the 2000 U.S. standard population, consistent with national mortality reporting methods.^[8]^ The annual AAMRs were calculated for the overall population and separately by sex, age group, race and ethnicity, urbanization category, census region, and state.

Secondary outcomes included the total number of CRC-related deaths and the distribution of deaths by location. The place-of-death frequencies were reported as proportions of total CRC-related deaths during the full study period.

### 2.4. Trend analysis

Temporal trends in CRC mortality from 1999 to 2025 were evaluated using the Joinpoint Regression Program. Annual AAMRs were modeled on a log-linear scale to identify statistically significant changes in mortality trends. The annual percent change (APC) and average annual percent change (AAPC) were calculated with the corresponding 95% confidence intervals (CIs).^[12,13]^

The inflection points were identified using permutation-based model selection. An increasing or decreasing APC or AAPC was defined as a slope significantly different from zero using a two-sided test at α = 0.05. Trend analyses were performed for the overall population and for each prespecified demographic and geographic stratum separately.

### 2.5. Forecasting analysis

Forecasting used a documented ensemble of 2 complementary univariate time-series models fitted independently to each stratum’s annual AAMR series from 1999 to 2025 (1999–2020 for urbanization). Annual forecasts were generated from 2026 to 2050, except for urbanization, for which the classification is available only through 2020, and therefore, the forecasts span 2021 to 2050.

The first ensemble member was an exponential-smoothing state-space model with additive errors and an additive damped trend (damped Holt method), which extrapolates the most recent local trend while damping it toward stability over time. The second was a nonseasonal autoregressive integrated moving-average model with first-order differencing (*d* = 1), with autoregressive and moving-average orders (*p* and *q*, each 0–2) selected by minimizing the Akaike information criterion. For each stratum, the 2 model forecasts were combined by inverse-error weighting, in which each model was weighted in proportion to the reciprocal of its mean absolute error in a rolling-origin, expanding-window, one-step-ahead backtest over the final 8 observed years (2018–2025 for most strata; 2013–2020 for urbanization). Ninety-five percent prediction intervals (PIs) were formed by combining the 2 models’ interval bounds with the same weights and were truncated at zero.

Forecast performance was assessed for each stratum using the backtest mean error, mean absolute error, and mean absolute percentage error (MAPE); bias was interpreted as low when MAPE was < 3%, moderate when 3 to 5%, and high when > 5% (Table S9, Supplemental Digital Content 1). All backtest MAPE values were <5%. Forecasts were computed in Python using the statsmodels library, and the analysis code is available from the corresponding author upon request. Forecasted AAMRs were reported overall and by sex, age group, race and ethnicity, urbanization category, and census region.

### 2.6. State-level analysis

State-level colorectal cancer mortality was analyzed for 2 periods: 1999 to 2020 and 2021 to 2025. The mean AAMRs were calculated for each state and the District of Columbia. The states were ranked according to the mean AAMR during each period. The highest-burden states were identified using the upper 90th percentile, and the lowest-burden states were identified using the lower 10th percentile. This approach was used to describe geographic variation and identify states with consistently high or low CRC mortality burdens.

### 2.7. Software and statistical analysis

Data preprocessing, descriptive analysis, model fitting, subgroup-specific forecasting, calibration assessment, and visualization were performed using the Python programming language. Joinpoint trend analyses were conducted using the National Cancer Institute Joinpoint Regression Program. All statistical tests were two-sided, and *P* < .05 was considered statistically significant. The observed and projected AAMRs are visualized using solid lines for historical trends and dashed lines for projected trends, with PIs displayed where applicable.

## 3. Results

### 3.1. Overall mortality burden and place of death

Between 1999 and 2025, 1,674,393 colorectal cancer-related deaths were recorded among U.S. adults aged ≥ 25 years. Men accounted for 873,839 deaths and women for 800,554 deaths (Table S1, Supplemental Digital Content 2).

Most deaths occurred at the decedent’s home (41.15%), followed by inpatient medical facilities (26.70%), nursing homes or long-term care facilities (16.58%), hospice facilities (7.77%), other settings (5.13%), outpatient or emergency medical facilities (2.18%), unknown place of death (0.23%), dead on arrival (0.18%), and medical facilities with unknown status (0.08%; Table S2, Supplemental Digital Content 3).

### 3.2. Overall trend and projection

The overall AAMR decreased from 38.11 per 100,000 in 1999 to 23.14 per 100,000 in 2025 (AAPC, –1.99; 95% CI, –2.13 to –1.85; *P* < .001; Fig. 1). Joinpoint analysis showed a significant decline from 1999 to 2016 (APC = –2.87; 95% CI = –3.01 to –2.74; *P* < .001), followed by a stable trend through 2025. The mean AAMR over the observation window was 28.05. Projections indicate a continued but slower decline, with the AAMR decreasing from 23.02 (95% PI, 22.09–23.95) in 2026 to 21.56 (95% PI, 9.97–33.15) by 2050 (Table 1 and Table S3, Supplemental Digital Content 4).

**Table 1:** Demographic characteristics of colorectal cancer-related number of deaths and average age-adjusted mortality rates (AAMR) per 100,000 population in adults aged ≥ 25 years in the United States from 1999 to 2025.

Demographics	Deaths	Mean age-adjusted mortality rate (95% CI)
Overall	1,674,393	28.05 (27.83–28.28)
Sex		
Women	800,554	23.43 (23.16–23.7)
Men	873,839	34.13 (33.75–34.51)
Race		
NH American Indian or Alaska Native	8839	25.9 (22.87–28.96)
NH Asian or Pacific Islander	45,892	18.56 (17.61–19.52)
NH Black or African American	209,148	37.45 (36.6–38.31)
NH White	1,304,861	28.07 (27.81–28.32)
Hispanic or Latino	105,653	21.03 (20.31–21.76)
Age group		
25–44 yrs	48,145	2.21 (2.11–2.31)
45–64 yrs	400,224	18.12 (17.83–18.42)
65–85 + yrs	1,226,024	106.48 (105.5–107.46)
Census region		
Northeast	323,634	27.93 (27.43–28.44)
Midwest	393,978	29.56 (29.08–30.04)
South	626,440	28.44 (28.07–28.81)
West	330,341	25.66 (25.2–26.12)
Urbanization		
Metropolitan	1,094,590	28.49 (28.24–28.74)
Nonmetropolitan	266,649	32.43 (31.84–33.01)

Deaths and mean AAMR span 1999–2025 for all strata except urbanization, which is classifiable only through 2020; therefore, the urbanization strata do not sum to the overall total.

AAMR = age-adjusted mortality rate per 100,000 population, CI = confidence interval, NH = non-Hispanic.

**Figure 1.**
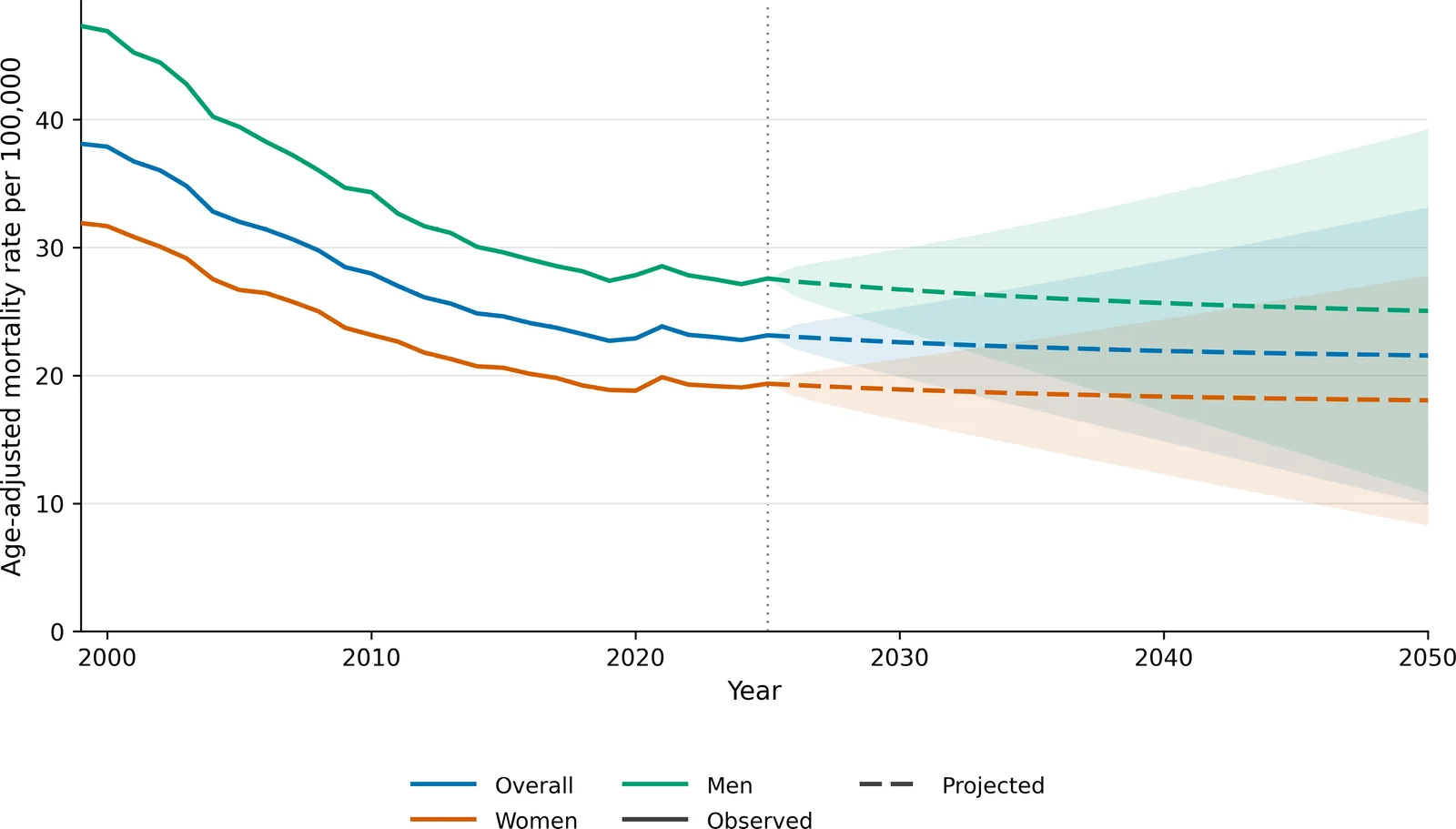
Age-adjusted colorectal cancer mortality rates, overall and by sex, in the United States, 1999–2050. Solid lines represent observed AAMRs per 100,000 population from 1999 to 2025; dashed lines represent projected AAMRs from 2026 to 2050. The shaded bands represent the 95% prediction intervals. The rates were standardized to the 2000 U.S. standard population. AAMR = age-adjusted mortality rate, U.S. = United States.

### 3.3. Stratified by sex

Both sexes showed a decline in colorectal cancer mortality during the observation period. Men had consistently higher AAMRs than women, with mean AAMRs of 34.13 and 23.43, respectively. Among women, the AAMR decreased significantly from 1999 to 2013 (APC, –2.99; 95% CI, –3.17 to –2.81; *P* < .001), continued to decline until 2019 (APC, –1.80; 95% CI, –2.74 to –0.86; *P* < .001), and then remained stable until 2025. The AAMR for women is projected to decline slightly from 19.26 in 2026 to 18.06 in 2050.

Among men, the AAMR decreased significantly from 1999 to 2015 (APC, –3.09; 95% CI, –3.23 to –2.96; *P* < .001), followed by a slower but still significant decline through 2025 (APC, –0.63; 95% CI, –0.92 to –0.35; *P* < .001). The men’s AAMR is projected to decrease from 27.35 in 2026 to 25.05 in 2050. The absolute sex gap is projected to narrow slightly over the projection period (Fig. 1; Table S1, Supplemental Digital Content 2; Table S3, Supplemental Digital Content 4).

### 3.4. Stratified by age

Colorectal cancer mortality was highest among adults aged 65 to 85 + years (mean AAMR, 106.48), followed by those aged 45 to 64 years (mean AAMR, 18.12) and 25 to 44 years (mean AAMR, 2.21). In the 25- to 44-year age group, the AAMR increased significantly across the full observation window from 1999 to 2025 (APC, 1.13; 95% CI, 0.93–1.33; *P* < .001). The AAMR in this group is projected to increase only slightly, from 2.69 in 2026 to 2.87 in 2050; the significant observed 1999 to 2025 increase (APC, 1.13) is the stronger signal, and the forecast is deliberately conservative.

Among adults aged 45 to 64 years, the AAMR was stable from 1999 to 2002, declined significantly from 2002 to 2005 (APC, –4.18; 95% CI, –7.69 to –0.54; *P* = .027), remained stable until 2017, and then increased significantly until 2025 (APC, 0.91; 95% CI, 0.53 to 1.30; *P* < .001). As with the 25 to 44 age group, this significant recent increase is the stronger near-term signal; the forecast’s near-flat trajectory, from 18.35 in 2026 to 18.33 in 2050, is deliberately conservative because it reflects the influence of the full 1999 to 2025 series rather than an expectation that the recent upward trend will reverse itself.

In adults aged 65 to 85 + years, the AAMR decreased significantly from 1999 to 2017 (APC, –3.48; 95% CI, –3.62 to –3.34; *P* < .001) and continued to decline until 2025 (APC, –0.75; 95% CI, –1.26 to –0.24; *P* = .006). The projected AAMR in this age group remained nearly stable, decreasing slightly from 79.59 in 2026 to 78.17 in 2050 (Fig. 2, Table S3, Supplemental Digital Content 4, Table S4, Supplemental Digital Content 5).

**Figure 2.**
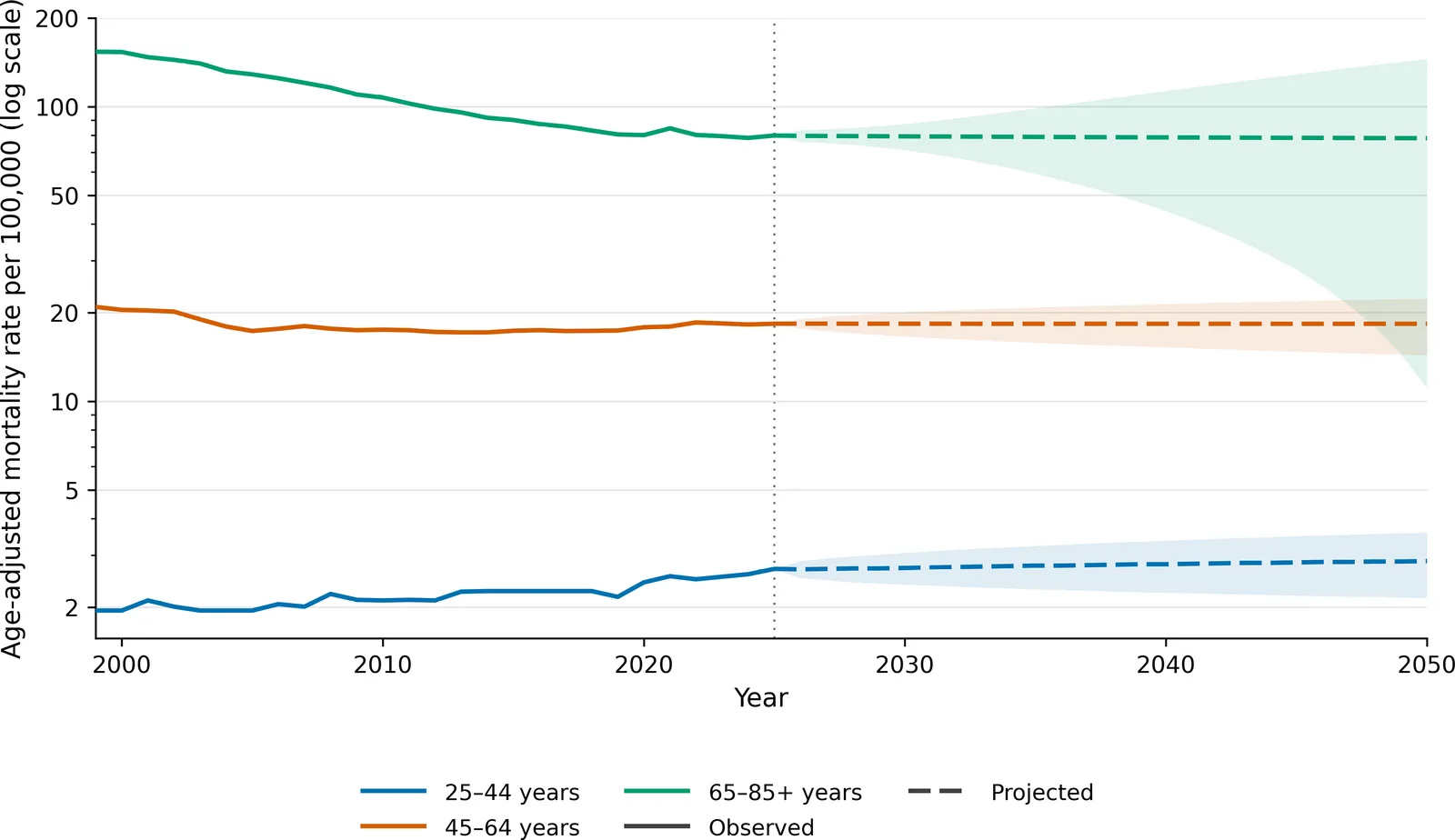
Age-adjusted colorectal cancer mortality rates by age group in the United States from 1999 to 2050. Solid lines represent observed AAMRs per 100,000 population from 1999 to 2025; dashed lines represent projected AAMRs from 2026 to 2050. The shaded bands represent the 95% prediction intervals. The rates were standardized to the 2000 U.S. standard population. The vertical axis uses a logarithmic scale so that all 3 age groups, whose rates differ by more than an order of magnitude, are legible. AAMR = age-adjusted mortality rate, U.S. = United States.

### 3.5. Stratified by race and ethnicity

The highest mean AAMR was observed among NH Black or African American adults (37.45), followed by NH White (28.07), NH American Indian or Alaska Native (25.90), Hispanic or Latino (21.03), and NH Asian or Pacific Islander (18.56). Temporal patterns differed across the racial and ethnic groups (Fig. 3, Table S5, Supplemental Digital Content 6).

**Figure 3.**
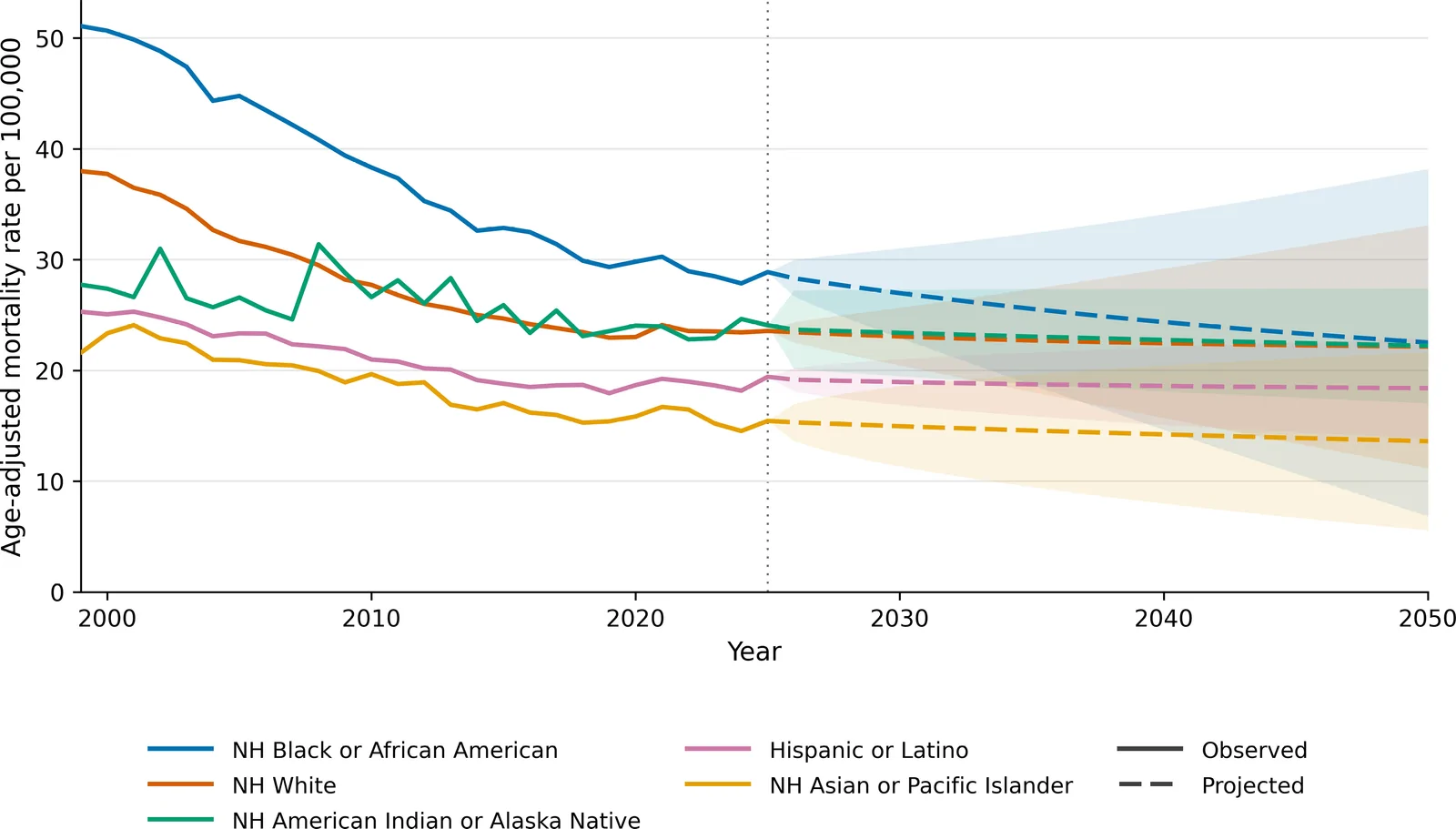
Age-adjusted colorectal cancer mortality rates by race and ethnicity in the United States from 1999 to 2050. Solid lines represent observed AAMRs per 100,000 population from 1999 to 2025; dashed lines represent projected AAMRs from 2026 to 2050. The shaded bands represent the 95% prediction intervals. The rates were standardized to the 2000 U.S. standard population. AAMR = age-adjusted mortality rate, NH = non-Hispanic, U.S. = United States.

Among Hispanic or Latino adults, the AAMR decreased significantly from 1999 to 2017 (APC = –1.91; 95% CI = –2.12 to –1.69; *P* < .001), followed by a stable trend until 2025. Among NH American Indian or Alaska Native adults, the AAMR declined significantly across the entire observation period from 1999 to 2025 (APC = –0.79; 95% CI = –1.12 to –0.47; *P* < .001). Among NH Asian or Pacific Islander adults, the AAMR decreased significantly from 1999 to 2017 (APC = –2.23; 95% CI = –2.65 to –1.81; *P* < .001), followed by stability through 2025.

Among NH Black or African American adults, the AAMR decreased significantly from 1999 to 2018 (APC, –2.89; 95% CI, –3.05 to –2.73; *P* < .001), followed by a slower decline through 2025 (APC, –0.87; 95% CI, –1.58 to –0.16; *P* = .018). Among NH White adults, the AAMR decreased significantly from 1999 to 2012 (APC, –3.01; 95% CI, –3.18 to –2.84; *P* < .001), continued to decline until 2019 (APC, –1.64; 95% CI, –2.22 to –1.05; *P* < .001), and then remained stable until 2025.

Projected trajectories through 2050 show continued declines across all racial and ethnic groups: NH Black or African American adults (28.32 in 2026 to 22.53 in 2050), NH White adults (23.46 to 22.16), NH American Indian or Alaska Native adults (23.69 to 22.24), Hispanic or Latino adults (19.17 to 18.40), and NH Asian or Pacific Islander adults (15.32 to 13.62). NH Black adults are projected to remain the highest-burden major group, although their absolute rate declines, and PIs were wide for the smaller American Indian or Alaska Native and Asian or Pacific Islander strata (Table 2, Fig. 3, and Table S3, Supplemental Digital Content 4).

**Table 2:** Annual percent change (APC) and average annual percent change (AAPC) in colorectal cancer-related mortality among adults aged ≥ 25 years in the United States, 1999–2025.

Year	APC (95% CI)	*P*-value for APC	AAPC (95% CI)	*P*-value for AAPC
Overall				
1999–2016	−2.87 (−3.01 to −2.74)	<.000001		
2016–2025	−0.29 (−0.65 to 0.07)	.108033		
1999–2025			−1.99 (−2.13 to −1.85)	<.000001
Sex				
Women				
1999–2013	−2.99 (−3.17 to −2.81)	<.000001		
2013–2019	−1.80 (−2.74 to −0.86)	.000776		
2019–2025	0.26 (−0.46 to 0.98)	.460849		
1999–2025			−1.98 (−2.25 to −1.71)	<.000001
Men				
1999–2015	−3.09 (−3.23 to −2.96)	<.000001		
2015–2025	−0.63 (−0.92 to −0.35)	.000125		
1999–2025			−2.15 (−2.28 to −2.03)	<.000001
Race				
NH American Indian or Alaska Native				
1999–2025	−0.79 (−1.12 to −0.47)	.000042	−0.79 (−1.12 to −0.47)	.000042
NH Asian or Pacific Islander				
1999–2017	−2.23 (−2.65 to −1.81)	<.000001		
2017–2025	−0.59 (−1.70 to 0.53)	.284780		
1999–2025			−1.73 (−2.15 to −1.30)	<.000001
NH Black or African American				
1999–2018	−2.89 (−3.05 to −2.73)	<.000001		
2018–2025	−0.87 (−1.58 to −0.16)	.018436		
1999–2025			−2.35 (−2.56 to −2.14)	<.000001
NH White				
1999–2012	−3.01 (−3.18 to −2.84)	<.000001		
2012–2019	−1.64 (−2.22 to −1.05)	.000015		
2019–2025	0.41 (−0.19 to 1.02)	.165417		
1999–2025			−1.86 (−2.07 to −1.65)	<.000001
Hispanic or Latino				
1999–2017	−1.91 (−2.12 to −1.69)	<.000001		
2017–2025	0.39 (−0.17 to 0.95)	.161910		
1999–2025			−1.21 (−1.42 to −0.99)	<.000001
Age group				
25–44 yrs				
1999–2025	1.13 (0.93 to 1.33)	<.000001	1.13 (0.93 to 1.33)	<.000001
45–64 yrs				
1999–2002	−1.47 (−3.35 to 0.45)	.123206		
2002–2005	−4.18 (−7.69 to −0.54)	.027248		
2005–2017	−0.14 (−0.37 to 0.10)	.245897		
2017–2025	0.91 (0.53 to 1.30)	.000115		
1999–2025			−0.45 (−0.91 to 0.03)	.063429
65–85 + yrs				
1999–2017	−3.48 (−3.62 to −3.34)	<.000001		
2017–2025	−0.75 (−1.26 to −0.24)	.005944		
1999–2025			−2.65 (−2.82 to −2.48)	<.000001
Census region				
Northeast				
1999–2012	−3.68 (−3.86 to −3.50)	<.000001		
2012–2019	−2.77 (−3.43 to −2.10)	<.000001		
2019–2025	−1.03 (−1.71 to −0.35)	.005338		
1999–2025			−2.83 (−3.07 to −2.59)	<.000001
Midwest				
1999–2016	−2.90 (−3.05 to −2.76)	<.000001		
2016–2025	−0.40 (−0.82 to 0.02)	.059922		
1999–2025			−2.04 (−2.21 to −1.88)	<.000001
South				
1999–2012	−2.66 (−2.90 to −2.43)	<.000001		
2012–2018	−1.57 (−2.59 to −0.54)	.004769		
2018–2025	0.35 (−0.26 to 0.96)	.245186		
1999–2025			−1.61 (−1.90 to −1.32)	<.000001
West				
1999–2016	−2.67 (−2.84 to −2.50)	<.000001		
2016–2025	−0.03 (−0.48 to 0.41)	.879849		
1999–2025			−1.76 (−1.94 to −1.58)	<.000001
Urbanization				
Metropolitan				
1999–2013	−3.14 (−3.29 to −3.00)	<.000001		
2013–2020	−1.83 (−2.25 to −1.41)	<.000001		
1999–2020			−2.71 (−2.86 to −2.55)	<.000001
Nonmetropolitan				
1999–2014	−2.20 (−2.37 to −2.03)	<.000001		
2014–2020	−0.66 (−1.37 to 0.05)	.067969		
1999–2020			−1.76 (−1.98 to −1.54)	<.000001

Urbanization trends cover 1999 to 2020 because the urban–rural classification is available only until 2020.

AAPC = average annual percent change, APC = annual percent change, CI = confidence interval, NH = non-Hispanic.

For Hispanic or Latino adults, the 2017 to 2025 segment was not statistically significant (APC, 0.39; 95% CI, –0.17 to 0.95; *P* = .16); therefore, the projected continued decline (19.17 in 2026 to 18.40 in 2050) reflects the earlier, larger, and statistically significant 1999 to 2017 decline (APC, –1.91), rather than a confirmed continuation of the most recent trend. As with the other strata noted above, this projection should be interpreted with caution.

### 3.6. Stratified by urbanization

Nonmetropolitan areas had a higher mean AAMR than metropolitan areas during the observation period (32.43 vs 28.49). Within metropolitan areas, the AAMR decreased significantly from 1999 to 2013 (APC, –3.14; 95% CI, –3.29 to –3.00; *P* < .001) and continued to decline until 2020 (APC, –1.83; 95% CI, –2.25 to –1.41; *P* < .001). The metropolitan AAMR is projected to decrease from 21.57 in 2021 to 15.30 by 2050.

Within nonmetropolitan areas, the AAMR decreased significantly from 1999 to 2014 (APC, –2.20; 95% CI, –2.37 to –2.03; *P* < .001), followed by a stable trend through 2020. Projections indicate a continued but slower decline, with the nonmetropolitan AAMR decreasing from 27.10 in 2021 to 23.84 by 2050. These projections suggest that the urban–rural mortality gap may widen from approximately 5.5 per 100,000 in 2021 to approximately 8.5 per 100,000 in 2050, because metropolitan rates are projected to fall faster than nonmetropolitan rates (Fig. 4, Table S3, Supplemental Digital Content 4, Table S6, Supplemental Digital Content 7).

**Figure 4.**
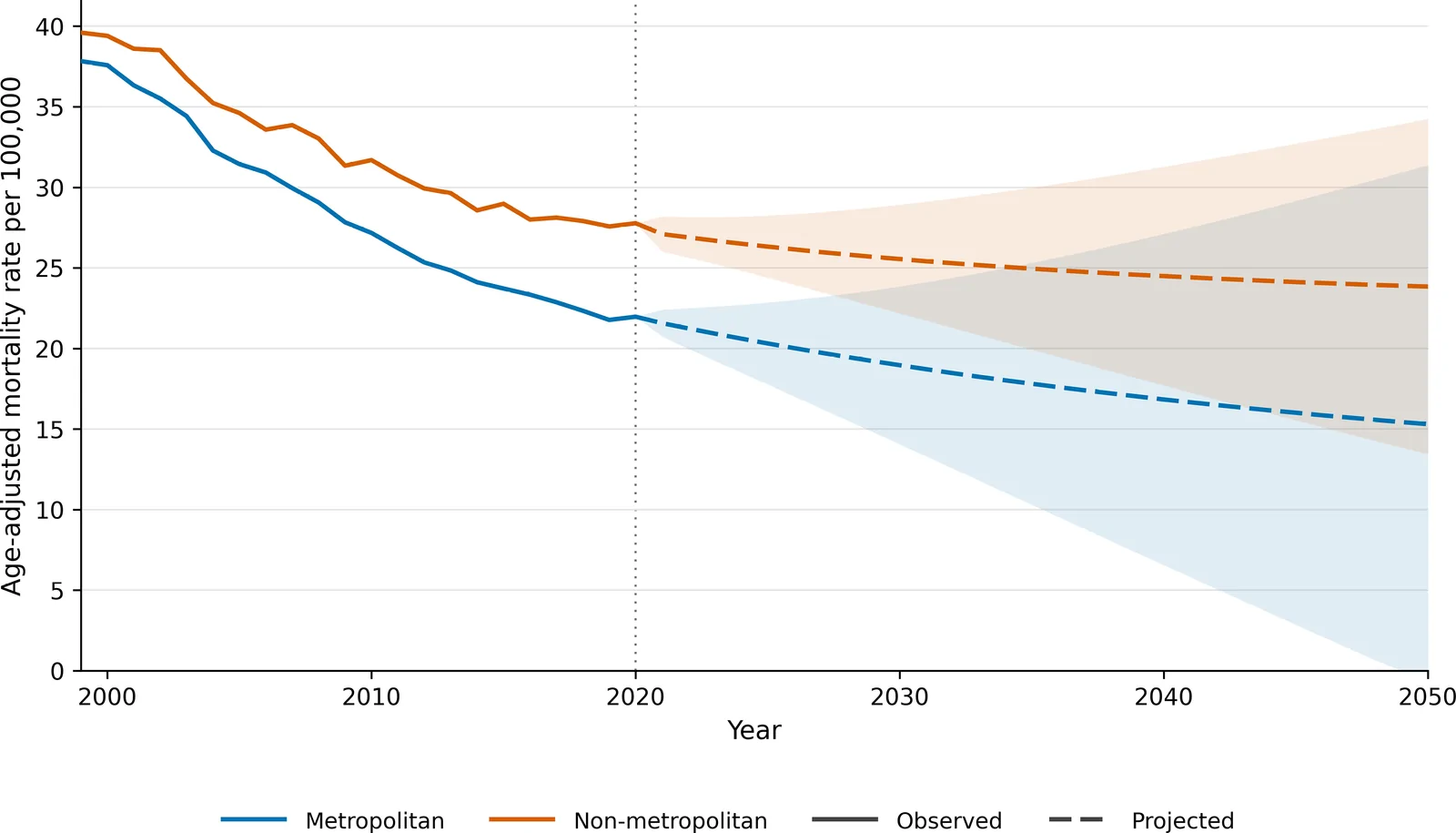
Age-adjusted colorectal cancer mortality rates by urbanization category in the United States, 1999–2050. Solid lines represent observed AAMRs per 100,000 population from 1999 to 2020; dashed lines represent projected AAMRs from 2021 to 2050. The shaded bands represent the 95% prediction intervals. Urbanization was classified according to the 2013 National Center for Health Statistics Urban–Rural Classification Scheme. The rates were standardized to the 2000 U.S. standard population. AAMR = age-adjusted mortality rate, U.S. = United States.

### 3.7. Stratified by census region

Across census regions, the Midwest had the highest mean AAMR (29.56), followed by the South (28.44), Northeast (27.93), and the West (25.66). In the Northeast, the AAMR decreased significantly from 1999 to 2012 (APC, –3.68; 95% CI, –3.86 to –3.50; *P* < .001), continued to decline through 2019 (APC, –2.77; 95% CI, –3.43 to –2.10; *P* < .001), and further declined through 2025 (APC, –1.03; 95% CI, –1.71 to –0.35; *P* = .005).

In the Midwest, the AAMR decreased significantly from 1999 to 2016 (APC, –2.90; 95% CI, –3.05 to –2.76; *P* < .001), followed by a stable trend until 2025. In the South, the AAMR decreased significantly from 1999 to 2012 (APC, –2.66; 95% CI, –2.90 to –2.43; *P* < .001), continued to decline through 2018 (APC, –1.57; 95% CI, –2.59 to –0.54; *P* = .005), and then remained stable until 2025. In the West, the AAMR decreased significantly from 1999 to 2016 (APC, –2.67; 95% CI, –2.84 to –2.50; *P* < .001), followed by stability through 2025.

Regional projections suggest modest divergence through 2050: the Northeast, which retained a significant recent decline, is projected to fall further (19.80 in 2026 to 15.57 in 2050), and the South is projected to decline slightly (24.44 to 23.18). The Midwest (24.13 to 25.08) and West (22.12 to 24.10) are projected to increase modestly because their most recent observed segments were flat and not statistically significant (Midwest APC, –0.40; 95% CI, –0.82 to 0.02; *P* = .06; West APC, –0.03; 95% CI, –0.48 to 0.41; *P* = .88). This projected increase reflects extrapolation over a 25-year horizon rather than a continuation of a statistically confirmed recent trend and should be interpreted with caution (Fig. 5, Table S3, Supplemental Digital Content 4, Table S7, Supplemental Digital Content 8).

**Figure 5.**
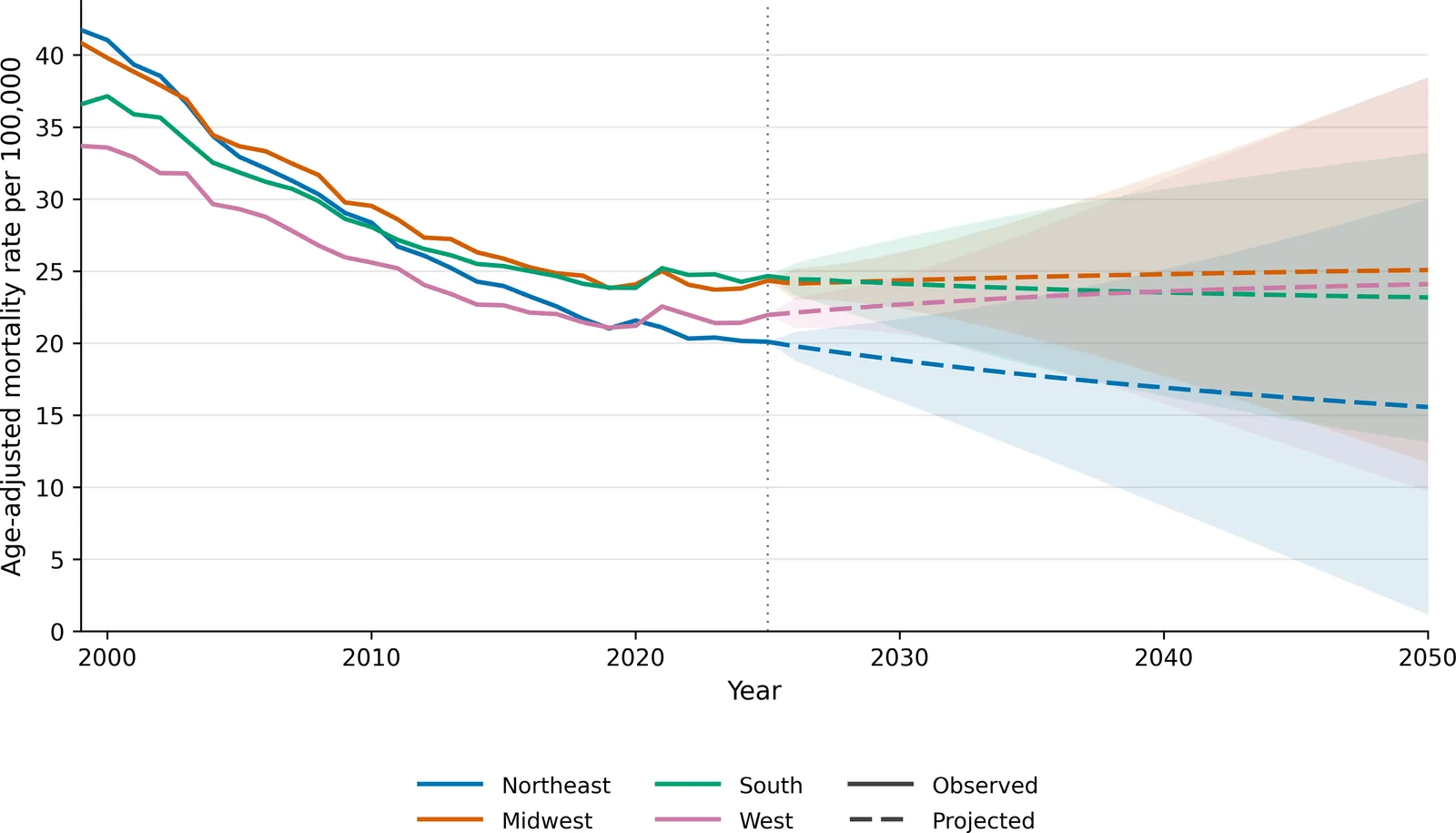
Age-adjusted colorectal cancer mortality rates by census region in the United States, 1999–2050. Solid lines represent observed AAMRs per 100,000 population from 1999 to 2025; dashed lines represent projected AAMRs from 2026 to 2050. Shaded bands represent the 95% prediction intervals. The census regions followed the standard U.S. Census Bureau’s definition. The rates were standardized to the 2000 U.S. standard population. AAMR = age-adjusted mortality rate, U.S. = United States.

### 3.8. Stratified by state

From 1999 to 2020, West Virginia had the highest mean state AAMR (35.82), and Utah had the lowest mean state AAMR (20.99). The states in the highest decile included West Virginia, Mississippi, the District of Columbia, Kentucky, and Nebraska, whereas those in the lowest decile included Utah, Arizona, Hawaii, Idaho, and Colorado.

From 2021 to 2025, Mississippi had the highest mean AAMR (33.11), and Massachusetts had the lowest mean AAMR (17.57). The states in the highest decile were Mississippi, Oklahoma, West Virginia, Kentucky, and Arkansas, whereas those in the lowest decile were Massachusetts, Connecticut, Utah, Hawaii, and New York (Table S8, Supplemental Digital Content 9).

## 4. Discussion

In this national analysis of CRC-related mortality among U.S. adults aged ≥ 25 years, 1,674,393 deaths were recorded between 1999 and 2025. The overall AAMR declined from 38.11 per 100,000 in 1999 to 23.14 per 100,000 in 2025. This decline is consistent with long-term national improvements in colorectal cancer screening, early detection, premalignant lesion removal, and treatment advances.^[1,2,14]^ Recent colorectal cancer statistics also show that mortality rates have decreased over time, although the pace of improvement has not been uniform across populations.^[15]^

However, the later stabilization of mortality and the projected continuation of only a slow decline from 2026 to 2050 suggest that previous mortality gains are decelerating rather than continuing at their earlier pace.^[14,15]^ This is the central finding of the present study: although CRC mortality has improved substantially over the past 2 decades, future progress may be limited if current prevention, diagnostic, and treatment systems remain unchanged.^[2,7]^

This projected deceleration of the mortality decline is clinically and public health relevant because CRC remains one of the most preventable malignancies when screening is completed and abnormal findings are followed by timely diagnostic colonoscopy.^[7,16]^ Current U.S. recommendations support average-risk colorectal cancer screening beginning at age 45, and modeling studies show that screening can reduce both incidence and mortality when adherence and follow-up are strong.^[16,17]^ However, evidence reviews have emphasized that screening benefits depend not only on eligibility but also on test completion, repeat adherence, and diagnostic colonoscopy after abnormal stool-based tests.^[18]^ Therefore, stable projected mortality should not be interpreted as adequate disease control. This should be interpreted as an indication that current colorectal cancer control strategies may not be sufficient to produce further meaningful mortality reductions.^[17,18]^

### 4.1. Persistent racial and ethnic disparities

The highest mean AAMR was observed among NH Black or African American adults, followed by NH White, NH American Indian or Alaska Native, Hispanic or Latino, and Asian or Pacific Islander adults. This pattern is consistent with prior evidence from the U.S. showing that colorectal cancer incidence, stage at diagnosis, subsite distribution, and survival vary substantially by race and ethnicity.^[4,19]^ Recent multi-cohort evidence also shows that racial disparities remain evident in colorectal cancer outcomes and access to care.^[20,21]^

Although mortality declined among NH Black adults during the observed period, their overall mortality burden remained the highest among all racial and ethnic groups. Therefore, a decline over time should not be misinterpreted as equity; a population can improve and still remain structurally disadvantaged.^[20,21]^ The persistence of racial differences is likely driven by unequal screening access, diagnostic delay, treatment quality, insurance coverage, comorbidity burden, socioeconomic disadvantage, and structural barriers to healthcare delivery.^[20,21]^ Biological and tumor-related differences may contribute to some variation, but the available evidence does not support explaining these disparities through biology alone.^[22]^

For NH Black adults, although the projected rate declines, it is projected to remain the highest among major racial and ethnic groups through 2050; therefore, passive guideline-based screening alone is unlikely to close the gap. More active interventions, including patient navigation, abnormal test tracking, timely colonoscopy completion, treatment delay monitoring, and access to high-quality oncology facilities, are needed to reduce the remaining mortality gap.^[20,21]^

The findings for NH American Indian or Alaska Native, Hispanic or Latino, and Asian or Pacific Islander populations require careful interpretation. American Indian and Alaska Native populations experience substantial cancer disparities, but national estimates can be distorted by racial misclassification, small annual counts, and regional heterogeneity.^[23]^ Hispanic adults with limited English proficiency and other access barriers may also have lower colorectal cancer screening completion, indicating that language access, insurance status, and healthcare navigation remain important determinants of prevention.^[24,25]^ Therefore, projected declines in smaller racial and ethnic groups should be treated as directional estimates rather than evidence that colorectal cancer disparities have been resolved.^[23–25]^

### 4.2. Urban–rural divergence

Nonmetropolitan areas had a higher average AAMR than metropolitan areas, indicating that rural populations continue to experience a disproportionate CRC mortality burden. This finding aligns with the National Center for Health Statistics urban–rural classification framework used in this analysis and with recent systematic review evidence showing rural–urban disparities in colorectal cancer screening, diagnosis, treatment, and survivorship care.^[11,26]^

Rural populations often have lower screening uptake, limited endoscopy capacity, longer travel distance, fewer oncology specialists, lower availability of high-volume cancer centers, and delayed treatment initiation, all of which can increase mortality risk.^[26,27]^ The projection pattern also warrants further attention. Metropolitan AAMRs are projected to decline faster than nonmetropolitan AAMRs; therefore, the absolute rural–urban gap is projected to widen through 2050 unless targeted interventions are implemented (Fig. 4, Table S3, Supplemental Digital Content 4, Table S6, Supplemental Digital Content 7).

Recent Behavioral Risk Factor Surveillance System-based evidence shows that rural–urban screening disparities remain substantial and are only partly explained by measurable factors such as education, income, and insurance status.^[28]^ This means that rural colorectal cancer mortality is not simply an individual behavior problem; it reflects deeper access barriers and health system design failures. Therefore, rural colorectal cancer control should include mailed fecal immunochemical test programs, patient navigation after abnormal tests, transportation support, regional endoscopy capacity, tele-oncology pathways, and referral systems that shorten the time from screening to diagnosis and treatment.^[26,28]^

### 4.3. Regional and state-level concentration of mortality

Regional analysis showed that the Midwest had the highest average AAMR, followed by the South, Northeast, and West. State-level findings further showed that West Virginia had the highest AAMR from 1999 to 2020, while Mississippi had the highest AAMR from 2021 to 2025. Other high-burden states included Kentucky, Ohio, Oklahoma, Arkansas, and the District of Columbia (Table S8, Supplemental Digital Content 9). These findings indicate that CRC mortality is geographically concentrated rather than evenly distributed across the U.S.

Prior U.S. cancer surveillance has shown that rural and urban populations differ in terms of cancer incidence and temporal trends.^[29]^ This concentration likely reflects overlapping structural risks, including rural residence, poverty, lower screening completion, higher obesity burden, limited specialist access, delayed diagnostic follow-up, and unequal treatment infrastructure.^[26,30]^ Recent evidence also links neighborhood socioeconomic disadvantage with colorectal cancer incidence and mortality risk.^[30]^ Therefore, high-burden states should be treated as priority settings for colorectal cancer control rather than simply being described as geographic outliers.

### 4.4. Sex differences and persistent male excess

Men had consistently higher CRC mortality than women throughout the study period, with a mean AAMR of 34.13 compared with 23.43 in women. Although mortality declined in both sexes, the male burden remained higher, and men are projected to continue having higher AAMRs through 2050. This finding is consistent with national colorectal cancer statistics showing persistent sex-based differences in the colorectal cancer burden.^[1,14,15]^

Sex- and gender-related factors may influence colorectal cancer risk, screening behavior, tumor characteristics, treatment toxicity, and outcomes.^[31,32]^ The male excess should not be interpreted as biology alone. Men may have higher exposure to modifiable risk factors, lower preventive healthcare use, delayed symptom evaluation, and different screening participation patterns.^[31]^ These findings support more direct male-focused prevention strategies, especially among middle-aged and older men in rural communities, high-burden states, and populations with limited preventive care engagement.^[31,32]^

### 4.5. Place of death and end-of-life care

Most CRC deaths occurred at home, followed by inpatient medical facilities, nursing homes or long-term care facilities, hospice facilities, and outpatient or emergency settings. This distribution indicates that a substantial share of terminal colorectal cancer care occurs outside inpatient acute care settings and highlights the relevance of home-based care, hospice availability, family caregiving, and palliative care services.^[33]^ Prior U.S. cancer studies have also shown that the place of death varies by race, age, geography, and access to hospice services.^[34]^

The high proportion of home deaths may reflect patient preference and broader shifts toward home-based end-of-life care,^[33,34]^ but the place of death alone should not be interpreted as a direct marker of care quality. Racial disparities in end-of-life care among patients with metastatic cancer have also been documented, reinforcing the need for equitable access to palliative care.^[35]^ The inpatient death burden remains substantial and may reflect differences in clinical needs, care preferences, access, and end-of-life care pathways.^[33,35]^

### 4.6. Pandemic-era disruption and post-2020 interpretation

The stabilization of mortality in recent years should be interpreted in the context of the COVID-19-related disruption of colorectal cancer screening and diagnostic services. A systematic review found that the pandemic substantially affected colorectal cancer screening across multiple settings.^[36]^ Modeling studies further suggest that delayed screening and disrupted care pathways may increase the future colorectal cancer burden if recovery is incomplete or if backlogs persist.^[37]^

This is important for the interpretation of projections. If missed screening and delayed colonoscopy are fully recovered, future mortality may decline more than projected.^[36,37]^ If backlogs persist or if abnormal stool tests are not followed by diagnostic colonoscopy, the mortality rate may exceed projections. The forecasting model cannot fully separate a genuine slowdown in the underlying decline from temporary, pandemic-related disruption. Therefore, the post-2020 period should be closely monitored as additional mortality data become available.

### 4.7. Younger-adult CRC mortality and early-onset context

The most notable age-specific finding was the significant increase in AAMR among adults aged 25 to 44 years between 1999 and 2025. Although the absolute mortality rate in this group remained much lower than that in older adults, the direction of change is important. This finding is consistent with prior evidence of the rising burden of early-onset colorectal cancer.^[3]^ Recent international data also show an increasing incidence of colorectal cancer among younger adults across multiple countries and territories.^[38]^

Therefore, the projected increase in the 25 to 44 age group should be regarded as a signal that warrants continued surveillance. Younger adults often fall outside traditional screening pathways unless they have high-risk features, and symptoms such as rectal bleeding, altered bowel habits, abdominal pain, unexplained anemia, or weight loss may be misattributed to benign conditions.^[3]^ Recent evidence also shows racial, ethnic, and socioeconomic variations in early-onset colorectal cancer survival, indicating that a younger age does not remove the influence of social determinants and access barriers.^[39,40]^

However, this finding should not be overstated. The largest absolute mortality burden remains in adults aged ≥ 65 years. The correct public health response is a dual strategy: preserve screening and treatment gains in older adults while improving symptom recognition, risk assessment, and diagnostic timeliness in younger adults.^[3,38]^

### 4.8. Forecast model interpretation and projection uncertainty

The projections suggest that overall CRC mortality may continue to slowly decline through 2050, more slowly than during 1999 to 2016, with meaningful differences across demographic and geographic strata. Because each stratum was modeled independently, subgroup forecasts need not sum exactly to the overall projection, and PIs were wide for small strata and lengthened over the 25-year horizon. However, forecasts are conditional estimates rather than fixed ones. For several strata (including the Midwest and West census regions, and, to a lesser degree, several sex and racial or ethnic subgroups), the most recent observed segment was flat or not statistically significant, yet the 25-year forecast trajectory still shows directional change; such projections are driven by curvature across the full 1999 to 2025 series rather than by a statistically confirmed recent trend and should be weighted less heavily than forecasts for strata with a significant recent trend in the same direction. Global colorectal and cancer burden projection studies have shown that future mortality is strongly shaped by age structure, population growth, risk factor prevalence, screening uptake, diagnostic access, and treatment availability.^[5,41,42]^

The use of CDC WONDER death certificate data provides national coverage but also requires cautious interpretation because mortality estimates depend on cause-of-death certification, coding accuracy, and population denominators.^[8]^ Similarly, the joinpoint-based trend interpretation depends on the observed annual AAMR series and model-selected inflection points.^[12,13]^ If prevention, screening completion, diagnostic follow-up, and treatment access improve, future mortality may fall below the projections.^[16–18]^ If current gaps persist, especially in rural, high-burden, and socioeconomically disadvantaged populations, the projected slow decline may underestimate future burden.^[26,30]^

### 4.9. Implications for colorectal cancer prevention and policy

These findings have several practical implications for colorectal cancer prevention and policy. First, screening expansion must be matched with screening completion. Lowering the screening age is useful only if eligible adults complete the screening and receive timely diagnostic colonoscopy after abnormal stool-based tests.^[2,16–18]^ Second, colorectal cancer control must be more geographically targeted. High-burden states and nonmetropolitan areas require stronger screening infrastructure, endoscopy access, referral networks, oncology capacity, and survivorship care pathways.^[26,29,30]^

Third, equity should be measured across the entire care pathway, not just by screening initiation. Health systems should track abnormal test follow-up, time to colonoscopy, stage at diagnosis, treatment initiation, guideline-concordant therapy, survival, palliative care access, and end-of-life outcomes.^[20,21]^ Fourth, prevention strategies must address modifiable risk factors, including excess body weight, poor diet, alcohol use, smoking, and physical inactivity, as these factors remain central to cancer prevention in the U.S.^[6,43]^

## 5. Future directions

Several research priorities emerge from these findings. First, subgroup-specific projections would benefit from linking CDC WONDER mortality data with richer individual-level data sources, including cancer registry data, Medicare claims, screening records, and treatment datasets. Such a linkage would help distinguish mortality changes driven by incidence, stage at diagnosis, screening history, treatment access, survival after diagnosis, and death certification practices.^[8,16,18]^ Mortality data alone cannot determine whether the observed deaths reflect higher disease occurrence, delayed diagnosis, incomplete treatment, poorer survival, competing mortality, or differences in how CRC is recorded on death certificates.^[8,9]^

Second, the persistent nonmetropolitan burden and state-level concentration of CRC mortality require replication and mechanistic investigation at finer geographical scales. County-level and regional analyses should examine screening completion, abnormal stool test follow-up, colonoscopy capacity, travel distance, oncology referral patterns, socioeconomic vulnerability, and treatment availability in high-burden states such as Mississippi, West Virginia, Kentucky, Oklahoma, Arkansas, and Ohio.^[26,29,30]^ Such work would clarify whether the projected slower rural decline reflects true deceleration, delayed access to screening and treatment, model behavior, or unmeasured structural barriers in local healthcare systems.^[26,28]^ Third, future studies should separate colon, rectosigmoid, and rectal cancers because the present ICD-10 definition combines C18–C20 into 1 outcome.^[9]^ Subsite-specific analyses are important because colon and rectal cancers may differ in age distribution, risk factors, screening detectability, treatment pathways, survival, and early-onset trends.^[38,44]^ Future work should also disaggregate racial and ethnic categories wherever possible, particularly for American Indian or Alaska Native, Hispanic or Latino, and Asian or Pacific Islander populations, because broad categories can hide clinically important subgroup differences.^[19,23–25]^ Fourth, early-onset CRC requires continuous surveillance. The rising mortality signal among adults aged 25 to 44 years should be evaluated using linked incidence, stage, symptom, diagnostic delay, and survival data to determine whether the increase reflects a true rising disease burden, later-stage presentation, delayed diagnosis, risk-factor shifts, or disparities in access to timely evaluation.^[3,38–40]^ Finally, projection models should be updated as post-2025 data become available, especially because pandemic-era screening disruptions may continue to affect the observed mortality trends and future estimates.^[36,37]^

## 6. Limitations

Several limitations should be considered when interpreting the findings of this study. We relied on death certificate data from CDC WONDER, which may be affected by misclassification of the cause of death, incomplete documentation, and changes in certification practices over time.^[8]^ Cause-of-death coding may not perfectly capture the contribution of CRC in complex cases.^[8,9]^ Although CDC WONDER provides the most complete national mortality surveillance data available for this type of analysis, it cannot fully separate true changes in CRC mortality from changes in coding, documentation, or certification behavior.^[8]^

The analysis is ecological; therefore, individual-level causal interpretations are not possible. We could not determine whether mortality changes were driven by changes in incidence, stage at diagnosis, screening history, diagnostic colonoscopy completion, treatment receipt, survival after diagnosis, comorbidity burden, insurance coverage, socioeconomic position, education, diet, obesity, alcohol use, smoking, family history, molecular tumor features, or healthcare facility quality.^[6,16–18]^ These unmeasured factors are central to colorectal cancer outcomes and may vary substantially across sex, age, racial and ethnic groups, urbanization categories, regions, and states.^[20,26,30]^

Race and ethnicity categories were based on death certificate classifications and may be subject to misclassification, particularly for American Indian or Alaska Native decedents.^[23]^ In addition, aggregate categories such as Asian or Pacific Islander and Hispanic or Latino combine heterogeneous populations with different ancestries, immigration histories, language access, socioeconomic conditions, screening behaviors, and healthcare access.^[19,24,25]^ Therefore, subgroup projections for these populations should be interpreted cautiously and should not be used to assume uniform risk or improvement within broad racial and ethnic groups.

CRC deaths were identified by any mention of ICD-10 codes C18–C20 on the death certificate rather than by the underlying cause alone; therefore, the counts and rates reported here include deaths in which CRC was a contributing cause and are correspondingly higher than estimates restricted to the underlying cause. The case definition used ICD-10 codes C18–C20, which appropriately capture malignant neoplasms of the colon, rectosigmoid junction, and rectum, but do not allow detailed interpretation by anatomic subsite within the present aggregate analysis.^[9]^ Colon and rectal cancers may have different epidemiology, screening pathways, treatment patterns, and survival profiles, particularly in early-onset disease.^[38,44]^ State-level analyses were also divided into 1999 to 2020 and 2021 to 2025, which allowed for a comparison of earlier and more recent mortality burdens, but limited continuous state-level trend modeling across the entire observation period.

The projection model was calibrated to observed 1999 to 2025 AAMRs and assumed broad continuity of the underlying processes. Therefore, projections should be interpreted as conditional forecasts rather than deterministic predictions.^[41,42]^ Because 2025 mortality data were provisional at the time of analysis, the terminal-year rate that anchors the forecasts may change as final data are released, and because strata were modeled independently, the subgroup forecasts need not sum exactly to the overall projection. Forecasts may be less stable in smaller subgroups, racial and ethnic groups with sparse annual counts, and strata affected by abrupt recent changes, as reflected by the wider PIs in some strata. Pandemic-era disruptions in screening, diagnosis, and treatment may also propagate into projections and contribute to unstable subgroup patterns.^[36,37]^ Finally, CDC WONDER does not capture several factors relevant to equity in colorectal cancer outcomes, including screening access, diagnostic delays, treatment quality, palliative care quality, caregiver burden, structural racism, language access, and sexual orientation or gender identity issues.

## 7. Conclusion

In conclusion, CRC mortality among U.S. adults aged ≥ 25 years declined substantially from 1999 to 2025, but the slowing pace of decline and the projected continuation of only a slow decline through 2050 indicate that the earlier, faster gains are not expected to resume without stronger intervention. The burden remains disproportionately concentrated among men, NH Black adults, nonmetropolitan populations, older adults, and residents of selected high-mortality states, while the increasing mortality trend among adults aged 25 to 44 years highlights the growing importance of early-onset CRC. These findings support the need for targeted colorectal cancer control strategies that strengthen screening completion, timely diagnostic colonoscopy, rural healthcare access, racial and geographic equity, risk factor prevention, and earlier integration of palliative and end-of-life care.

## Acknowledgments

The APC was covered by Alfaisal University.

## Author contributions

**Conceptualization:** Kanza Atif, Sadia Qazi.

**Data curation:** Kanza Atif, Vishan Das, Sadia Qazi, Mazhar Ali.

**Formal analysis:** Kanza Atif.

**Investigation:** Eshal Atif.

**Methodology:** Kanza Atif, Eshal Atif, Vishan Das, Abdal Ahmad.

**Project administration:** Eshal Atif.

**Supervision:** Vishan Das, Sadia Qazi, Akef Obeidat.

**Validation:** Muhammad Atif Mazhar.

**Visualization:** Mohib Ali Hassan, Muhammad Mukhlis.

**Writing – original draft:** Kanza Atif, Eshal Atif, Vishan Das, Sadia Qazi, Mazhar Ali, Abdal Ahmad, Fnu Urooba, Mohib Ali Hassan, Muhammad Mukhlis, Muhammad Atif Mazhar, Akef Obeidat.

**Writing – review & editing:** Kanza Atif, Eshal Atif, Vishan Das, Sadia Qazi, Mazhar Ali, Abdal Ahmad, Fnu Urooba, Mohib Ali Hassan, Muhammad Mukhlis, Muhammad Atif Mazhar, Akef Obeidat.


















