# Supplementary material for: Trends and projections of colorectal carcinoma mortality in U.S. adults, 1999–2050: A CDC WONDER-based ecological time-series analysis
Source: Medicine (Baltimore). 2026 Oct 16;105(42):e51146. doi: 10.1097/MD.0000000000051146 (PMC13681118; doi:10.1097/MD.0000000000051146)
Supplement: Supplementary file 1 [file medi-105-e51146-s001.docx]

**Trends and Projections of Colorectal Carcinoma Mortality in U.S. Adults, 1999–2050: A CDC WONDER-Based Ecological Time-Series Analysis**

Supplemental Table 9: Forecast accuracy by stratum, from a rolling-origin (expanding-window) one-step-ahead backtest over the final eight observed years (2018–2025; 2013–2020 for urbanization). MAE, mean absolute error (per 100,000); MAPE, mean absolute percentage error. Forecast bias: low <3%, moderate 3–5%, high >5%.

| Stratification | Backtest MAE | Backtest MAPE (%) | Forecast bias |
| --- | --- | --- | --- |
| Overall | 0.47 | 2.02 | Low |
| Women | 0.44 | 2.25 | Low |
| Men | 0.54 | 1.93 | Low |
| NH American Indian or Alaska Native | 0.94 | 3.98 | Moderate |
| NH Asian or Pacific Islander | 0.65 | 4.19 | Moderate |
| NH Black or African American | 0.82 | 2.79 | Low |
| NH White | 0.40 | 1.68 | Low |
| Hispanic or Latino | 0.63 | 3.36 | Moderate |
| 25-44 Years | 0.10 | 3.95 | Moderate |
| 45-64 Years | 0.24 | 1.32 | Low |
| 65-85+ Years | 2.23 | 2.74 | Low |
| Northeast | 0.42 | 1.98 | Low |
| Midwest | 0.61 | 2.53 | Low |
| South | 0.52 | 2.12 | Low |
| West | 0.54 | 2.45 | Low |
| Metropolitan | 0.36 | 1.57 | Low |
| Non-metropolitan | 0.48 | 1.70 | Low |
