# Supplementary material for: Trends and projections of colorectal carcinoma mortality in U.S. adults, 1999–2050: A CDC WONDER-based ecological time-series analysis
Source: Medicine (Baltimore). 2026 Oct 16;105(42):e51146. doi: 10.1097/MD.0000000000051146 (PMC13681118; doi:10.1097/MD.0000000000051146)
Supplement: Supplementary file 2 [file medi-105-e51146-s002.docx]

**Trends and Projections of Colorectal Carcinoma Mortality in U.S. Adults, 1999–2050: A CDC WONDER-Based Ecological Time-Series Analysis**

**Supplemental Table 1:** Trends in Age-Adjusted Mortality Rates and Deaths from Colorectal cancer among United States Adults Aged 25 and Older stratified by Overall and Sex, 1999–2025.

| **Year** | **Overall** | | **Women** | | **Men** | |
| --- | --- | --- | --- | --- | --- | --- |
|  | **Deaths** | **AAMR (95% CI)** | **Deaths** | **AAMR (95% CI)** | **Deaths** | **AAMR (95% CI)** |
| **1999** | 67334 | 38.11 (37.82–38.4) | 33754 | 31.91 (31.57–32.25) | 33580 | 47.32 (46.8–47.83) |
| **2000** | 67766 | 37.88 (37.6–38.17) | 33955 | 31.67 (31.33–32.01) | 33811 | 46.91 (46.4–47.42) |
| **2001** | 66790 | 36.73 (36.45–37.01) | 33422 | 30.83 (30.49–31.16) | 33368 | 45.24 (44.75–45.74) |
| **2002** | 66616 | 36.03 (35.76–36.31) | 33022 | 30.07 (29.74–30.39) | 33594 | 44.46 (43.97–44.94) |
| **2003** | 65467 | 34.81 (34.54–35.08) | 32501 | 29.15 (28.83–29.47) | 32966 | 42.77 (42.3–43.24) |
| **2004** | 62653 | 32.81 (32.55–33.07) | 31062 | 27.52 (27.21–27.82) | 31591 | 40.22 (39.76–40.67) |
| **2005** | 62299 | 32.01 (31.76–32.26) | 30574 | 26.68 (26.38–26.98) | 31725 | 39.43 (38.99–39.87) |
| **2006** | 62393 | 31.42 (31.17–31.67) | 30771 | 26.45 (26.15–26.75) | 31622 | 38.27 (37.84–38.7) |
| **2007** | 62172 | 30.66 (30.42–30.9) | 30488 | 25.77 (25.47–26.06) | 31684 | 37.24 (36.82–37.65) |
| **2008** | 61587 | 29.77 (29.53–30) | 30076 | 25.01 (24.73–25.3) | 31511 | 36.03 (35.62–36.43) |
| **2009** | 60133 | 28.47 (28.24–28.69) | 29009 | 23.73 (23.45–24) | 31124 | 34.67 (34.27–35.06) |
| **2010** | 60127 | 27.97 (27.74–28.19) | 28726 | 23.17 (22.9–23.44) | 31401 | 34.32 (33.94–34.71) |
| **2011** | 59646 | 27 (26.78–27.22) | 28705 | 22.65 (22.39–22.92) | 30941 | 32.66 (32.29–33.03) |
| **2012** | 59054 | 26.11 (25.9–26.33) | 28139 | 21.79 (21.53–22.05) | 30915 | 31.67 (31.31–32.03) |
| **2013** | 59231 | 25.62 (25.41–25.83) | 27989 | 21.29 (21.03–21.54) | 31242 | 31.14 (30.78–31.49) |
| **2014** | 58750 | 24.85 (24.65–25.06) | 27754 | 20.72 (20.47–20.97) | 30996 | 30.05 (29.71–30.39) |
| **2015** | 59611 | 24.62 (24.42–24.83) | 28147 | 20.6 (20.36–20.85) | 31464 | 29.62 (29.28–29.95) |
| **2016** | 59519 | 24.1 (23.9–24.3) | 27897 | 20.13 (19.89–20.37) | 31622 | 29.06 (28.73–29.39) |
| **2017** | 59812 | 23.73 (23.54–23.92) | 27997 | 19.81 (19.57–20.05) | 31815 | 28.55 (28.23–28.88) |
| **2018** | 59728 | 23.23 (23.04–23.42) | 27690 | 19.22 (18.98–19.45) | 32038 | 28.14 (27.82–28.45) |
| **2019** | 59555 | 22.71 (22.52–22.9) | 27582 | 18.87 (18.64–19.09) | 31973 | 27.4 (27.1–27.71) |
| **2020** | 60996 | 22.9 (22.71–23.08) | 27842 | 18.81 (18.59–19.04) | 33154 | 27.84 (27.53–28.15) |
| **2021** | 61727 | 23.83 (23.64–24.02) | 28279 | 19.87 (19.64–20.11) | 33448 | 28.54 (28.23–28.86) |
| **2022** | 62129 | 23.18 (23–23.37) | 28527 | 19.29 (19.06–19.52) | 33602 | 27.83 (27.53–28.14) |
| **2023** | 62119 | 23 (22.82–23.19) | 28377 | 19.17 (18.94–19.4) | 33742 | 27.51 (27.21–27.81) |
| **2024** | 63043 | 22.77 (22.59–22.95) | 28891 | 19.06 (18.84–19.29) | 34152 | 27.14 (26.85–27.44) |
| **2025** | 64136 | 23.14 (22.96–23.33) | 29378 | 19.36 (19.13–19.59) | 34758 | 27.58 (27.28–27.88) |
| **Total/Average** | 1674393 | 28.05 (27.83–28.28) | 800554 | 23.43 (23.16–23.7) | 873839 | 34.13 (33.75–34.51) |

*AAMR, age-adjusted mortality rate per 100,000 population; CI, confidence interval.*
