# Supplementary material for: Trends and projections of colorectal carcinoma mortality in U.S. adults, 1999–2050: A CDC WONDER-based ecological time-series analysis
Source: Medicine (Baltimore). 2026 Oct 16;105(42):e51146. doi: 10.1097/MD.0000000000051146 (PMC13681118; doi:10.1097/MD.0000000000051146)
Supplement: Supplementary file 3 [file medi-105-e51146-s003.docx]

**Trends and Projections of Colorectal Carcinoma Mortality in U.S. Adults, 1999–2050: A CDC WONDER-Based Ecological Time-Series Analysis**

**Supplemental Table 2:** Percentage distribution of place of death in the United States (1999–2025), with Colorectal cancer related mortality highlighted.

| **Place of Death** | **Deaths** | **% Total** |
| --- | --- | --- |
| **Medical Facility - Inpatient** | 447,011 | 26.70% |
| **Medical Facility - Outpatient or ER** | 36,577 | 2.18% |
| **Medical Facility - Dead on Arrival** | 3,015 | 0.18% |
| **Medical Facility - Status unknown** | 1,275 | 0.08% |
| **Decedent's home** | 689,023 | 41.15% |
| **Hospice facility** | 130,057 | 7.77% |
| **Nursing home/long term care** | 277,693 | 16.58% |
| **Other** | 85,968 | 5.13% |
| **Place of death unknown** | 3,774 | 0.23% |
| **Total** | 1,674,393 | 100% |
