# Supplementary material for: Trends and projections of colorectal carcinoma mortality in U.S. adults, 1999–2050: A CDC WONDER-based ecological time-series analysis
Source: Medicine (Baltimore). 2026 Oct 16;105(42):e51146. doi: 10.1097/MD.0000000000051146 (PMC13681118; doi:10.1097/MD.0000000000051146)
Supplement: Supplementary file 4 [file medi-105-e51146-s004.docx]

**Trends and Projections of Colorectal Carcinoma Mortality in U.S. Adults, 1999–2050: A CDC WONDER-Based Ecological Time-Series Analysis**

**Supplemental Table 3: Colorectal cancer related Forecast Age-Adjusted Mortality Rates, Stratified by Overall, Sex, Race, Age, Census region, and Urbanization in Adults in the United States, 2026 to 2050. Forecasts are from a damped-trend exponential smoothing and ARIMA ensemble (see Methods); 95% prediction intervals are shown and were truncated at zero. Intervals are wide for the smaller American Indian or Alaska Native and Asian or Pacific Islander strata and widen over the horizon.**

| **Stratification** | **Year** | **Forecast Value (95% PI)** |
| --- | --- | --- |
| Overall | 2026 | 23.02 (22.09–23.95) |
| Overall | 2027 | 22.91 (21.49–24.32) |
| Overall | 2028 | 22.80 (20.94–24.65) |
| Overall | 2029 | 22.70 (20.42–24.98) |
| Overall | 2030 | 22.60 (19.90–25.31) |
| Overall | 2031 | 22.52 (19.39–25.64) |
| Overall | 2032 | 22.43 (18.88–25.99) |
| Overall | 2033 | 22.35 (18.37–26.34) |
| Overall | 2034 | 22.28 (17.86–26.70) |
| Overall | 2035 | 22.21 (17.36–27.06) |
| Overall | 2036 | 22.15 (16.86–27.44) |
| Overall | 2037 | 22.09 (16.35–27.82) |
| Overall | 2038 | 22.03 (15.85–28.21) |
| Overall | 2039 | 21.97 (15.35–28.60) |
| Overall | 2040 | 21.92 (14.85–29.00) |
| Overall | 2041 | 21.88 (14.36–29.40) |
| Overall | 2042 | 21.83 (13.86–29.80) |
| Overall | 2043 | 21.79 (13.37–30.21) |
| Overall | 2044 | 21.75 (12.88–30.63) |
| Overall | 2045 | 21.71 (12.39–31.04) |
| Overall | 2046 | 21.68 (11.90–31.46) |
| Overall | 2047 | 21.65 (11.41–31.88) |
| Overall | 2048 | 21.62 (10.93–32.30) |
| Overall | 2049 | 21.59 (10.45–32.73) |
| Overall | 2050 | 21.56 (9.97–33.15) |
| **Sex** | | |
| Women | 2026 | 19.26 (18.41–20.11) |
| Women | 2027 | 19.16 (17.88–20.44) |
| Women | 2028 | 19.07 (17.41–20.74) |
| Women | 2029 | 18.99 (16.95–21.03) |
| Women | 2030 | 18.91 (16.51–21.31) |
| Women | 2031 | 18.84 (16.08–21.60) |
| Women | 2032 | 18.77 (15.65–21.89) |
| Women | 2033 | 18.71 (15.22–22.19) |
| Women | 2034 | 18.64 (14.80–22.49) |
| Women | 2035 | 18.59 (14.37–22.80) |
| Women | 2036 | 18.53 (13.95–23.12) |
| Women | 2037 | 18.49 (13.53–23.44) |
| Women | 2038 | 18.44 (13.12–23.76) |
| Women | 2039 | 18.39 (12.70–24.09) |
| Women | 2040 | 18.35 (12.29–24.42) |
| Women | 2041 | 18.31 (11.88–24.75) |
| Women | 2042 | 18.28 (11.47–25.09) |
| Women | 2043 | 18.25 (11.06–25.43) |
| Women | 2044 | 18.21 (10.66–25.77) |
| Women | 2045 | 18.18 (10.25–26.11) |
| Women | 2046 | 18.16 (9.85–26.46) |
| Women | 2047 | 18.13 (9.45–26.81) |
| Women | 2048 | 18.11 (9.06–27.15) |
| Women | 2049 | 18.08 (8.66–27.50) |
| Women | 2050 | 18.06 (8.27–27.85) |
| Men | 2026 | 27.35 (26.21–28.49) |
| Men | 2027 | 27.18 (25.49–28.86) |
| Men | 2028 | 27.02 (24.83–29.21) |
| Men | 2029 | 26.87 (24.18–29.55) |
| Men | 2030 | 26.72 (23.54–29.91) |
| Men | 2031 | 26.59 (22.90–30.27) |
| Men | 2032 | 26.46 (22.27–30.65) |
| Men | 2033 | 26.34 (21.63–31.04) |
| Men | 2034 | 26.22 (21.00–31.45) |
| Men | 2035 | 26.12 (20.36–31.87) |
| Men | 2036 | 26.01 (19.73–32.30) |
| Men | 2037 | 25.92 (19.09–32.75) |
| Men | 2038 | 25.83 (18.45–33.20) |
| Men | 2039 | 25.74 (17.82–33.67) |
| Men | 2040 | 25.66 (17.18–34.14) |
| Men | 2041 | 25.58 (16.55–34.62) |
| Men | 2042 | 25.51 (15.91–35.11) |
| Men | 2043 | 25.44 (15.28–35.61) |
| Men | 2044 | 25.38 (14.64–36.11) |
| Men | 2045 | 25.32 (14.01–36.62) |
| Men | 2046 | 25.26 (13.38–37.14) |
| Men | 2047 | 25.20 (12.75–37.66) |
| Men | 2048 | 25.15 (12.12–38.18) |
| Men | 2049 | 25.10 (11.49–38.71) |
| Men | 2050 | 25.05 (10.86–39.24) |
| **Race** | | |
| NH American Indian or Alaska Native | 2026 | 23.69 (20.14–27.24) |
| NH American Indian or Alaska Native | 2027 | 23.61 (19.97–27.26) |
| NH American Indian or Alaska Native | 2028 | 23.54 (19.80–27.28) |
| NH American Indian or Alaska Native | 2029 | 23.47 (19.64–27.30) |
| NH American Indian or Alaska Native | 2030 | 23.40 (19.48–27.31) |
| NH American Indian or Alaska Native | 2031 | 23.33 (19.33–27.32) |
| NH American Indian or Alaska Native | 2032 | 23.26 (19.18–27.33) |
| NH American Indian or Alaska Native | 2033 | 23.19 (19.04–27.34) |
| NH American Indian or Alaska Native | 2034 | 23.13 (18.90–27.35) |
| NH American Indian or Alaska Native | 2035 | 23.06 (18.77–27.36) |
| NH American Indian or Alaska Native | 2036 | 23.00 (18.63–27.36) |
| NH American Indian or Alaska Native | 2037 | 22.94 (18.50–27.37) |
| NH American Indian or Alaska Native | 2038 | 22.88 (18.38–27.37) |
| NH American Indian or Alaska Native | 2039 | 22.82 (18.25–27.38) |
| NH American Indian or Alaska Native | 2040 | 22.76 (18.13–27.38) |
| NH American Indian or Alaska Native | 2041 | 22.70 (18.02–27.39) |
| NH American Indian or Alaska Native | 2042 | 22.64 (17.90–27.39) |
| NH American Indian or Alaska Native | 2043 | 22.59 (17.79–27.39) |
| NH American Indian or Alaska Native | 2044 | 22.54 (17.67–27.40) |
| NH American Indian or Alaska Native | 2045 | 22.48 (17.57–27.40) |
| NH American Indian or Alaska Native | 2046 | 22.43 (17.46–27.41) |
| NH American Indian or Alaska Native | 2047 | 22.38 (17.35–27.41) |
| NH American Indian or Alaska Native | 2048 | 22.33 (17.25–27.42) |
| NH American Indian or Alaska Native | 2049 | 22.28 (17.15–27.42) |
| NH American Indian or Alaska Native | 2050 | 22.24 (17.05–27.42) |
| NH Asian or Pacific Islander | 2026 | 15.32 (13.64–16.99) |
| NH Asian or Pacific Islander | 2027 | 15.23 (12.91–17.55) |
| NH Asian or Pacific Islander | 2028 | 15.14 (12.32–17.96) |
| NH Asian or Pacific Islander | 2029 | 15.06 (11.81–18.30) |
| NH Asian or Pacific Islander | 2030 | 14.97 (11.35–18.60) |
| NH Asian or Pacific Islander | 2031 | 14.89 (10.93–18.85) |
| NH Asian or Pacific Islander | 2032 | 14.81 (10.54–19.09) |
| NH Asian or Pacific Islander | 2033 | 14.74 (10.17–19.30) |
| NH Asian or Pacific Islander | 2034 | 14.66 (9.82–19.50) |
| NH Asian or Pacific Islander | 2035 | 14.58 (9.49–19.68) |
| NH Asian or Pacific Islander | 2036 | 14.51 (9.17–19.86) |
| NH Asian or Pacific Islander | 2037 | 14.44 (8.86–20.02) |
| NH Asian or Pacific Islander | 2038 | 14.37 (8.56–20.18) |
| NH Asian or Pacific Islander | 2039 | 14.30 (8.27–20.33) |
| NH Asian or Pacific Islander | 2040 | 14.23 (8.00–20.47) |
| NH Asian or Pacific Islander | 2041 | 14.17 (7.73–20.61) |
| NH Asian or Pacific Islander | 2042 | 14.10 (7.46–20.74) |
| NH Asian or Pacific Islander | 2043 | 14.04 (7.21–20.87) |
| NH Asian or Pacific Islander | 2044 | 13.97 (6.96–20.99) |
| NH Asian or Pacific Islander | 2045 | 13.91 (6.72–21.11) |
| NH Asian or Pacific Islander | 2046 | 13.85 (6.48–21.23) |
| NH Asian or Pacific Islander | 2047 | 13.79 (6.25–21.34) |
| NH Asian or Pacific Islander | 2048 | 13.74 (6.02–21.45) |
| NH Asian or Pacific Islander | 2049 | 13.68 (5.80–21.56) |
| NH Asian or Pacific Islander | 2050 | 13.62 (5.58–21.67) |
| NH Black or African American | 2026 | 28.32 (26.67–29.97) |
| NH Black or African American | 2027 | 27.97 (25.65–30.28) |
| NH Black or African American | 2028 | 27.63 (24.72–30.54) |
| NH Black or African American | 2029 | 27.30 (23.82–30.78) |
| NH Black or African American | 2030 | 26.98 (22.94–31.03) |
| NH Black or African American | 2031 | 26.68 (22.08–31.28) |
| NH Black or African American | 2032 | 26.39 (21.23–31.54) |
| NH Black or African American | 2033 | 26.10 (20.38–31.82) |
| NH Black or African American | 2034 | 25.83 (19.55–32.11) |
| NH Black or African American | 2035 | 25.56 (18.72–32.41) |
| NH Black or African American | 2036 | 25.31 (17.89–32.72) |
| NH Black or African American | 2037 | 25.06 (17.07–33.05) |
| NH Black or African American | 2038 | 24.82 (16.26–33.39) |
| NH Black or African American | 2039 | 24.59 (15.45–33.74) |
| NH Black or African American | 2040 | 24.37 (14.65–34.10) |
| NH Black or African American | 2041 | 24.16 (13.85–34.47) |
| NH Black or African American | 2042 | 23.95 (13.05–34.85) |
| NH Black or African American | 2043 | 23.75 (12.26–35.24) |
| NH Black or African American | 2044 | 23.56 (11.47–35.64) |
| NH Black or African American | 2045 | 23.37 (10.69–36.05) |
| NH Black or African American | 2046 | 23.19 (9.91–36.46) |
| NH Black or African American | 2047 | 23.01 (9.14–36.89) |
| NH Black or African American | 2048 | 22.85 (8.37–37.32) |
| NH Black or African American | 2049 | 22.68 (7.61–37.76) |
| NH Black or African American | 2050 | 22.53 (6.85–38.20) |
| NH White | 2026 | 23.46 (22.55–24.36) |
| NH White | 2027 | 23.35 (21.97–24.72) |
| NH White | 2028 | 23.25 (21.45–25.05) |
| NH White | 2029 | 23.16 (20.95–25.36) |
| NH White | 2030 | 23.07 (20.46–25.68) |
| NH White | 2031 | 22.99 (19.97–26.00) |
| NH White | 2032 | 22.91 (19.49–26.33) |
| NH White | 2033 | 22.84 (19.01–26.67) |
| NH White | 2034 | 22.78 (18.54–27.01) |
| NH White | 2035 | 22.71 (18.06–27.36) |
| NH White | 2036 | 22.66 (17.59–27.72) |
| NH White | 2037 | 22.60 (17.12–28.08) |
| NH White | 2038 | 22.55 (16.65–28.45) |
| NH White | 2039 | 22.51 (16.19–28.83) |
| NH White | 2040 | 22.46 (15.72–29.20) |
| NH White | 2041 | 22.42 (15.26–29.59) |
| NH White | 2042 | 22.38 (14.80–29.97) |
| NH White | 2043 | 22.35 (14.34–30.36) |
| NH White | 2044 | 22.31 (13.88–30.75) |
| NH White | 2045 | 22.28 (13.43–31.14) |
| NH White | 2046 | 22.25 (12.97–31.53) |
| NH White | 2047 | 22.23 (12.52–31.93) |
| NH White | 2048 | 22.20 (12.08–32.33) |
| NH White | 2049 | 22.18 (11.63–32.72) |
| NH White | 2050 | 22.16 (11.19–33.12) |
| Hispanic or Latino | 2026 | 19.17 (18.12–20.22) |
| Hispanic or Latino | 2027 | 19.11 (17.73–20.50) |
| Hispanic or Latino | 2028 | 19.06 (17.41–20.71) |
| Hispanic or Latino | 2029 | 19.01 (17.13–20.88) |
| Hispanic or Latino | 2030 | 18.96 (16.88–21.04) |
| Hispanic or Latino | 2031 | 18.91 (16.65–21.17) |
| Hispanic or Latino | 2032 | 18.87 (16.43–21.30) |
| Hispanic or Latino | 2033 | 18.83 (16.23–21.42) |
| Hispanic or Latino | 2034 | 18.79 (16.05–21.53) |
| Hispanic or Latino | 2035 | 18.75 (15.87–21.64) |
| Hispanic or Latino | 2036 | 18.72 (15.70–21.74) |
| Hispanic or Latino | 2037 | 18.69 (15.54–21.84) |
| Hispanic or Latino | 2038 | 18.66 (15.38–21.93) |
| Hispanic or Latino | 2039 | 18.63 (15.23–22.02) |
| Hispanic or Latino | 2040 | 18.60 (15.09–22.11) |
| Hispanic or Latino | 2041 | 18.57 (14.95–22.20) |
| Hispanic or Latino | 2042 | 18.55 (14.82–22.28) |
| Hispanic or Latino | 2043 | 18.53 (14.69–22.36) |
| Hispanic or Latino | 2044 | 18.51 (14.57–22.45) |
| Hispanic or Latino | 2045 | 18.49 (14.45–22.53) |
| Hispanic or Latino | 2046 | 18.47 (14.33–22.60) |
| Hispanic or Latino | 2047 | 18.45 (14.22–22.68) |
| Hispanic or Latino | 2048 | 18.43 (14.11–22.76) |
| Hispanic or Latino | 2049 | 18.42 (14.00–22.83) |
| Hispanic or Latino | 2050 | 18.40 (13.89–22.91) |
| **Age group** | | |
| 25-44 Years | 2026 | 2.69 (2.51–2.87) |
| 25-44 Years | 2027 | 2.70 (2.47–2.93) |
| 25-44 Years | 2028 | 2.71 (2.43–2.98) |
| 25-44 Years | 2029 | 2.71 (2.41–3.02) |
| 25-44 Years | 2030 | 2.72 (2.39–3.06) |
| 25-44 Years | 2031 | 2.73 (2.37–3.10) |
| 25-44 Years | 2032 | 2.74 (2.35–3.14) |
| 25-44 Years | 2033 | 2.75 (2.33–3.17) |
| 25-44 Years | 2034 | 2.76 (2.32–3.20) |
| 25-44 Years | 2035 | 2.77 (2.30–3.23) |
| 25-44 Years | 2036 | 2.77 (2.29–3.26) |
| 25-44 Years | 2037 | 2.78 (2.28–3.29) |
| 25-44 Years | 2038 | 2.79 (2.26–3.32) |
| 25-44 Years | 2039 | 2.80 (2.25–3.34) |
| 25-44 Years | 2040 | 2.80 (2.24–3.37) |
| 25-44 Years | 2041 | 2.81 (2.23–3.39) |
| 25-44 Years | 2042 | 2.82 (2.22–3.42) |
| 25-44 Years | 2043 | 2.83 (2.21–3.44) |
| 25-44 Years | 2044 | 2.83 (2.20–3.46) |
| 25-44 Years | 2045 | 2.84 (2.19–3.49) |
| 25-44 Years | 2046 | 2.85 (2.18–3.51) |
| 25-44 Years | 2047 | 2.85 (2.17–3.53) |
| 25-44 Years | 2048 | 2.86 (2.17–3.55) |
| 25-44 Years | 2049 | 2.86 (2.16–3.57) |
| 25-44 Years | 2050 | 2.87 (2.15–3.59) |
| 45-64 Years | 2026 | 18.35 (17.68–19.02) |
| 45-64 Years | 2027 | 18.35 (17.29–19.40) |
| 45-64 Years | 2028 | 18.34 (17.02–19.67) |
| 45-64 Years | 2029 | 18.34 (16.79–19.89) |
| 45-64 Years | 2030 | 18.34 (16.59–20.08) |
| 45-64 Years | 2031 | 18.34 (16.41–20.26) |
| 45-64 Years | 2032 | 18.33 (16.25–20.42) |
| 45-64 Years | 2033 | 18.33 (16.10–20.57) |
| 45-64 Years | 2034 | 18.33 (15.95–20.71) |
| 45-64 Years | 2035 | 18.33 (15.82–20.84) |
| 45-64 Years | 2036 | 18.33 (15.69–20.96) |
| 45-64 Years | 2037 | 18.33 (15.57–21.08) |
| 45-64 Years | 2038 | 18.33 (15.46–21.20) |
| 45-64 Years | 2039 | 18.33 (15.35–21.31) |
| 45-64 Years | 2040 | 18.33 (15.24–21.42) |
| 45-64 Years | 2041 | 18.33 (15.14–21.52) |
| 45-64 Years | 2042 | 18.33 (15.04–21.62) |
| 45-64 Years | 2043 | 18.33 (14.94–21.71) |
| 45-64 Years | 2044 | 18.33 (14.84–21.81) |
| 45-64 Years | 2045 | 18.33 (14.75–21.90) |
| 45-64 Years | 2046 | 18.33 (14.66–21.99) |
| 45-64 Years | 2047 | 18.33 (14.57–22.08) |
| 45-64 Years | 2048 | 18.33 (14.49–22.16) |
| 45-64 Years | 2049 | 18.33 (14.40–22.25) |
| 45-64 Years | 2050 | 18.33 (14.32–22.33) |
| 65-85+ Years | 2026 | 79.59 (75.87–83.31) |
| 65-85+ Years | 2027 | 79.54 (75.18–83.89) |
| 65-85+ Years | 2028 | 79.47 (74.23–84.70) |
| 65-85+ Years | 2029 | 79.39 (72.90–85.88) |
| 65-85+ Years | 2030 | 79.31 (71.21–87.42) |
| 65-85+ Years | 2031 | 79.24 (69.20–89.27) |
| 65-85+ Years | 2032 | 79.17 (66.96–91.38) |
| 65-85+ Years | 2033 | 79.10 (64.51–93.68) |
| 65-85+ Years | 2034 | 79.03 (61.92–96.14) |
| 65-85+ Years | 2035 | 78.96 (59.19–98.73) |
| 65-85+ Years | 2036 | 78.90 (56.35–101.44) |
| 65-85+ Years | 2037 | 78.83 (53.42–104.24) |
| 65-85+ Years | 2038 | 78.77 (50.42–107.12) |
| 65-85+ Years | 2039 | 78.71 (47.35–110.08) |
| 65-85+ Years | 2040 | 78.65 (44.22–113.09) |
| 65-85+ Years | 2041 | 78.60 (41.04–116.16) |
| 65-85+ Years | 2042 | 78.55 (37.81–119.28) |
| 65-85+ Years | 2043 | 78.49 (34.55–122.43) |
| 65-85+ Years | 2044 | 78.44 (31.26–125.62) |
| 65-85+ Years | 2045 | 78.39 (27.94–128.84) |
| 65-85+ Years | 2046 | 78.35 (24.60–132.09) |
| 65-85+ Years | 2047 | 78.30 (21.24–135.36) |
| 65-85+ Years | 2048 | 78.25 (17.87–138.64) |
| 65-85+ Years | 2049 | 78.21 (14.48–141.94) |
| 65-85+ Years | 2050 | 78.17 (11.09–145.25) |
| **Census region** | | |
| Northeast | 2026 | 19.80 (18.83–20.77) |
| Northeast | 2027 | 19.54 (18.08–21.00) |
| Northeast | 2028 | 19.29 (17.37–21.21) |
| Northeast | 2029 | 19.05 (16.66–21.44) |
| Northeast | 2030 | 18.82 (15.95–21.68) |
| Northeast | 2031 | 18.59 (15.24–21.94) |
| Northeast | 2032 | 18.38 (14.53–22.22) |
| Northeast | 2033 | 18.17 (13.82–22.53) |
| Northeast | 2034 | 17.97 (13.09–22.85) |
| Northeast | 2035 | 17.78 (12.37–23.19) |
| Northeast | 2036 | 17.59 (11.64–23.55) |
| Northeast | 2037 | 17.42 (10.91–23.92) |
| Northeast | 2038 | 17.24 (10.17–24.31) |
| Northeast | 2039 | 17.08 (9.43–24.72) |
| Northeast | 2040 | 16.92 (8.69–25.14) |
| Northeast | 2041 | 16.76 (7.95–25.57) |
| Northeast | 2042 | 16.61 (7.20–26.02) |
| Northeast | 2043 | 16.47 (6.45–26.48) |
| Northeast | 2044 | 16.33 (5.70–26.95) |
| Northeast | 2045 | 16.19 (4.95–27.43) |
| Northeast | 2046 | 16.06 (4.19–27.93) |
| Northeast | 2047 | 15.93 (3.44–28.43) |
| Northeast | 2048 | 15.81 (2.68–28.94) |
| Northeast | 2049 | 15.69 (1.92–29.46) |
| Northeast | 2050 | 15.57 (1.16–29.99) |
| Midwest | 2026 | 24.13 (23.06–25.20) |
| Midwest | 2027 | 24.19 (23.03–25.35) |
| Midwest | 2028 | 24.25 (22.91–25.59) |
| Midwest | 2029 | 24.30 (22.70–25.91) |
| Midwest | 2030 | 24.36 (22.42–26.30) |
| Midwest | 2031 | 24.41 (22.08–26.74) |
| Midwest | 2032 | 24.46 (21.70–27.22) |
| Midwest | 2033 | 24.51 (21.28–27.73) |
| Midwest | 2034 | 24.55 (20.84–28.27) |
| Midwest | 2035 | 24.60 (20.36–28.83) |
| Midwest | 2036 | 24.64 (19.87–29.40) |
| Midwest | 2037 | 24.68 (19.36–30.00) |
| Midwest | 2038 | 24.72 (18.83–30.60) |
| Midwest | 2039 | 24.75 (18.29–31.22) |
| Midwest | 2040 | 24.79 (17.73–31.85) |
| Midwest | 2041 | 24.82 (17.16–32.48) |
| Midwest | 2042 | 24.86 (16.59–33.13) |
| Midwest | 2043 | 24.89 (16.00–33.78) |
| Midwest | 2044 | 24.92 (15.41–34.43) |
| Midwest | 2045 | 24.95 (14.81–35.10) |
| Midwest | 2046 | 24.98 (14.20–35.76) |
| Midwest | 2047 | 25.01 (13.58–36.43) |
| Midwest | 2048 | 25.03 (12.96–37.10) |
| Midwest | 2049 | 25.06 (12.34–37.78) |
| Midwest | 2050 | 25.08 (11.71–38.46) |
| South | 2026 | 24.44 (23.33–25.55) |
| South | 2027 | 24.41 (22.81–26.01) |
| South | 2028 | 24.28 (22.12–26.44) |
| South | 2029 | 24.22 (21.57–26.87) |
| South | 2030 | 24.12 (20.97–27.28) |
| South | 2031 | 24.06 (20.44–27.68) |
| South | 2032 | 23.98 (19.90–28.06) |
| South | 2033 | 23.91 (19.39–28.43) |
| South | 2034 | 23.85 (18.90–28.79) |
| South | 2035 | 23.79 (18.44–29.14) |
| South | 2036 | 23.73 (17.98–29.47) |
| South | 2037 | 23.67 (17.55–29.79) |
| South | 2038 | 23.62 (17.14–30.10) |
| South | 2039 | 23.57 (16.74–30.40) |
| South | 2040 | 23.52 (16.35–30.70) |
| South | 2041 | 23.48 (15.98–30.98) |
| South | 2042 | 23.44 (15.62–31.25) |
| South | 2043 | 23.40 (15.27–31.52) |
| South | 2044 | 23.36 (14.94–31.78) |
| South | 2045 | 23.33 (14.62–32.04) |
| South | 2046 | 23.29 (14.30–32.29) |
| South | 2047 | 23.26 (14.00–32.53) |
| South | 2048 | 23.23 (13.70–32.77) |
| South | 2049 | 23.21 (13.42–33.00) |
| South | 2050 | 23.18 (13.14–33.23) |
| West | 2026 | 22.12 (21.08–23.16) |
| West | 2027 | 22.27 (21.06–23.48) |
| West | 2028 | 22.41 (21.00–23.83) |
| West | 2029 | 22.55 (20.86–24.24) |
| West | 2030 | 22.68 (20.65–24.71) |
| West | 2031 | 22.80 (20.35–25.25) |
| West | 2032 | 22.91 (19.99–25.83) |
| West | 2033 | 23.02 (19.57–26.46) |
| West | 2034 | 23.12 (19.11–27.12) |
| West | 2035 | 23.21 (18.62–27.80) |
| West | 2036 | 23.30 (18.10–28.50) |
| West | 2037 | 23.38 (17.55–29.21) |
| West | 2038 | 23.46 (16.99–29.93) |
| West | 2039 | 23.53 (16.41–30.65) |
| West | 2040 | 23.60 (15.82–31.38) |
| West | 2041 | 23.66 (15.23–32.10) |
| West | 2042 | 23.73 (14.62–32.83) |
| West | 2043 | 23.78 (14.01–33.55) |
| West | 2044 | 23.84 (13.40–34.27) |
| West | 2045 | 23.89 (12.79–34.98) |
| West | 2046 | 23.93 (12.17–35.69) |
| West | 2047 | 23.98 (11.56–36.40) |
| West | 2048 | 24.02 (10.94–37.10) |
| West | 2049 | 24.06 (10.33–37.79) |
| West | 2050 | 24.10 (9.72–38.47) |
| **Urbanization** | | |
| Metropolitan | 2021 | 21.57 (20.74–22.40) |
| Metropolitan | 2022 | 21.24 (19.98–22.50) |
| Metropolitan | 2023 | 20.92 (19.24–22.60) |
| Metropolitan | 2024 | 20.61 (18.51–22.71) |
| Metropolitan | 2025 | 20.31 (17.77–22.85) |
| Metropolitan | 2026 | 20.02 (17.03–23.01) |
| Metropolitan | 2027 | 19.74 (16.29–23.19) |
| Metropolitan | 2028 | 19.47 (15.55–23.39) |
| Metropolitan | 2029 | 19.21 (14.80–23.61) |
| Metropolitan | 2030 | 18.96 (14.06–23.85) |
| Metropolitan | 2031 | 18.71 (13.31–24.11) |
| Metropolitan | 2032 | 18.47 (12.56–24.39) |
| Metropolitan | 2033 | 18.24 (11.81–24.68) |
| Metropolitan | 2034 | 18.02 (11.05–24.99) |
| Metropolitan | 2035 | 17.81 (10.30–25.31) |
| Metropolitan | 2036 | 17.60 (9.55–25.65) |
| Metropolitan | 2037 | 17.40 (8.80–26.00) |
| Metropolitan | 2038 | 17.21 (8.05–26.36) |
| Metropolitan | 2039 | 17.02 (7.30–26.73) |
| Metropolitan | 2040 | 16.83 (6.56–27.11) |
| Metropolitan | 2041 | 16.66 (5.81–27.50) |
| Metropolitan | 2042 | 16.49 (5.07–27.90) |
| Metropolitan | 2043 | 16.32 (4.33–28.31) |
| Metropolitan | 2044 | 16.16 (3.59–28.73) |
| Metropolitan | 2045 | 16.01 (2.85–29.16) |
| Metropolitan | 2046 | 15.85 (2.12–29.59) |
| Metropolitan | 2047 | 15.71 (1.39–30.03) |
| Metropolitan | 2048 | 15.57 (0.66–30.48) |
| Metropolitan | 2049 | 15.43 (0.00–30.93) |
| Metropolitan | 2050 | 15.30 (0.00–31.38) |
| Non-metropolitan | 2021 | 27.10 (26.02–28.19) |
| Non-metropolitan | 2022 | 26.89 (25.64–28.14) |
| Non-metropolitan | 2023 | 26.69 (25.24–28.14) |
| Non-metropolitan | 2024 | 26.50 (24.82–28.18) |
| Non-metropolitan | 2025 | 26.32 (24.39–28.25) |
| Non-metropolitan | 2026 | 26.15 (23.95–28.34) |
| Non-metropolitan | 2027 | 25.98 (23.51–28.46) |
| Non-metropolitan | 2028 | 25.83 (23.06–28.60) |
| Non-metropolitan | 2029 | 25.68 (22.62–28.75) |
| Non-metropolitan | 2030 | 25.55 (22.17–28.92) |
| Non-metropolitan | 2031 | 25.41 (21.72–29.11) |
| Non-metropolitan | 2032 | 25.29 (21.27–29.31) |
| Non-metropolitan | 2033 | 25.17 (20.82–29.52) |
| Non-metropolitan | 2034 | 25.06 (20.37–29.75) |
| Non-metropolitan | 2035 | 24.95 (19.92–29.98) |
| Non-metropolitan | 2036 | 24.85 (19.47–30.22) |
| Non-metropolitan | 2037 | 24.75 (19.03–30.47) |
| Non-metropolitan | 2038 | 24.66 (18.58–30.73) |
| Non-metropolitan | 2039 | 24.57 (18.14–31.00) |
| Non-metropolitan | 2040 | 24.49 (17.70–31.27) |
| Non-metropolitan | 2041 | 24.41 (17.26–31.55) |
| Non-metropolitan | 2042 | 24.33 (16.83–31.83) |
| Non-metropolitan | 2043 | 24.26 (16.40–32.12) |
| Non-metropolitan | 2044 | 24.19 (15.97–32.41) |
| Non-metropolitan | 2045 | 24.12 (15.54–32.71) |
| Non-metropolitan | 2046 | 24.06 (15.11–33.01) |
| Non-metropolitan | 2047 | 24.00 (14.69–33.31) |
| Non-metropolitan | 2048 | 23.94 (14.27–33.62) |
| Non-metropolitan | 2049 | 23.89 (13.85–33.93) |
| Non-metropolitan | 2050 | 23.84 (13.44–34.24) |

*AAMR, age-adjusted mortality rate per 100,000 population; PI, prediction interval.*
