# Supplementary material for: Trends and projections of colorectal carcinoma mortality in U.S. adults, 1999–2050: A CDC WONDER-based ecological time-series analysis
Source: Medicine (Baltimore). 2026 Oct 16;105(42):e51146. doi: 10.1097/MD.0000000000051146 (PMC13681118; doi:10.1097/MD.0000000000051146)
Supplement: Supplementary file 5 [file medi-105-e51146-s005.docx]

**Trends and Projections of Colorectal Carcinoma Mortality in U.S. Adults, 1999–2050: A CDC WONDER-Based Ecological Time-Series Analysis**

**Supplemental Table 4:** Trends in Age-Adjusted Mortality Rates and Deaths from Colorectal cancer among United States Adults Aged 25 and Older stratified by Age group, 1999–2025.

| **Year** | **25-44 Years** | | **45-64 Years** | | **65-85+ Years** | |
| --- | --- | --- | --- | --- | --- | --- |
|  | **Deaths** | **AAMR (95% CI)** | **Deaths** | **AAMR (95% CI)** | **Deaths** | **AAMR (95% CI)** |
| **1999** | 1658 | 1.95 (1.86–2.05) | 12650 | 20.91 (20.54–21.27) | 53026 | 153.63 (152.32–154.94) |
| **2000** | 1649 | 1.95 (1.86–2.05) | 12668 | 20.44 (20.08–20.79) | 53449 | 153.3 (152–154.6) |
| **2001** | 1728 | 2.11 (2.01–2.21) | 13084 | 20.34 (19.99–20.69) | 51978 | 147.22 (145.95–148.49) |
| **2002** | 1694 | 2.01 (1.91–2.1) | 13548 | 20.16 (19.82–20.5) | 51374 | 144.19 (142.95–145.44) |
| **2003** | 1599 | 1.95 (1.86–2.05) | 13283 | 18.98 (18.65–19.3) | 50585 | 140.16 (138.94–141.38) |
| **2004** | 1581 | 1.95 (1.86–2.05) | 13001 | 17.94 (17.63–18.25) | 48071 | 131.73 (130.56–132.91) |
| **2005** | 1591 | 1.95 (1.85–2.04) | 12995 | 17.33 (17.03–17.63) | 47713 | 128.72 (127.57–129.88) |
| **2006** | 1651 | 2.05 (1.96–2.15) | 13686 | 17.63 (17.34–17.93) | 47056 | 124.93 (123.8–126.06) |
| **2007** | 1589 | 2.01 (1.91–2.11) | 14382 | 18.01 (17.72–18.31) | 46201 | 120.49 (119.39–121.59) |
| **2008** | 1737 | 2.22 (2.11–2.32) | 14398 | 17.64 (17.35–17.93) | 45452 | 116.08 (115.01–117.15) |
| **2009** | 1649 | 2.12 (2.02–2.22) | 14604 | 17.44 (17.16–17.73) | 43880 | 110 (108.96–111.03) |
| **2010** | 1639 | 2.11 (2.01–2.21) | 14971 | 17.51 (17.23–17.79) | 43517 | 107.37 (106.35–108.38) |
| **2011** | 1667 | 2.12 (2.02–2.22) | 15282 | 17.43 (17.15–17.71) | 42697 | 102.53 (101.56–103.51) |
| **2012** | 1642 | 2.11 (2.01–2.21) | 15132 | 17.2 (16.92–17.47) | 42280 | 98.42 (97.47–99.36) |
| **2013** | 1754 | 2.26 (2.16–2.37) | 15203 | 17.14 (16.86–17.42) | 42274 | 95.63 (94.71–96.55) |
| **2014** | 1738 | 2.27 (2.17–2.38) | 15361 | 17.14 (16.86–17.41) | 41651 | 91.69 (90.8–92.58) |
| **2015** | 1774 | 2.27 (2.17–2.38) | 15708 | 17.36 (17.08–17.63) | 42129 | 90.13 (89.26–91) |
| **2016** | 1801 | 2.27 (2.17–2.38) | 15890 | 17.44 (17.16–17.71) | 41828 | 87.32 (86.47–88.16) |
| **2017** | 1794 | 2.27 (2.17–2.38) | 15829 | 17.32 (17.05–17.6) | 42189 | 85.61 (84.78–86.43) |
| **2018** | 1839 | 2.27 (2.17–2.38) | 15748 | 17.35 (17.07–17.62) | 42141 | 83 (82.2–83.8) |
| **2019** | 1768 | 2.17 (2.07–2.27) | 15772 | 17.39 (17.11–17.66) | 42015 | 80.52 (79.75–81.3) |
| **2020** | 1975 | 2.43 (2.32–2.53) | 16167 | 17.84 (17.56–18.12) | 42854 | 80.08 (79.31–80.84) |
| **2021** | 2048 | 2.55 (2.44–2.66) | 16147 | 17.94 (17.66–18.23) | 43532 | 84.4 (83.6–85.2) |
| **2022** | 2025 | 2.49 (2.38–2.6) | 16370 | 18.52 (18.23–18.82) | 43734 | 80.18 (79.43–80.94) |
| **2023** | 2086 | 2.54 (2.43–2.65) | 16183 | 18.38 (18.09–18.68) | 43850 | 79.42 (78.67–80.17) |
| **2024** | 2182 | 2.59 (2.48–2.7) | 16014 | 18.23 (17.94–18.52) | 44847 | 78.35 (77.63–79.09) |
| **2025** | 2287 | 2.7 (2.59–2.81) | 16148 | 18.34 (18.05–18.64) | 45701 | 79.82 (79.08–80.56) |
| **Total/Average** | 48145 | 2.21 (2.11–2.31) | 400224 | 18.12 (17.83–18.42) | 1226024 | 106.48 (105.5–107.46) |

*AAMR, age-adjusted mortality rate per 100,000 population; CI, confidence interval.*
