# Supplementary material for: Trends and projections of colorectal carcinoma mortality in U.S. adults, 1999–2050: A CDC WONDER-based ecological time-series analysis
Source: Medicine (Baltimore). 2026 Oct 16;105(42):e51146. doi: 10.1097/MD.0000000000051146 (PMC13681118; doi:10.1097/MD.0000000000051146)
Supplement: Supplementary file 6 [file medi-105-e51146-s006.docx]

**Trends and Projections of Colorectal Carcinoma Mortality in U.S. Adults, 1999–2050: A CDC WONDER-Based Ecological Time-Series Analysis**

**Supplemental Table 5:** Trends in Age-Adjusted Mortality Rates and Deaths from Colorectal cancer among United States Adults Aged 25 and Older stratified by Race, 1999–2025.

| **Year** | **NH American Indian or Alaska Native** | | **NH Asian or Pacific Islander** | | **NH Black or African American** | | **NH White** | | **Hispanic or Latino** | |
| --- | --- | --- | --- | --- | --- | --- | --- | --- | --- | --- |
|  | **Deaths** | **AAMR (95% CI)** | **Deaths** | **AAMR (95% CI)** | **Deaths** | **AAMR (95% CI)** | **Deaths** | **AAMR (95% CI)** | **Deaths** | **AAMR (95% CI)** |
| **1999** | 200 | 27.74 (23.64–31.83) | 923 | 21.6 (20.12–23.07) | 7657 | 51.08 (49.92–52.24) | 56281 | 37.99 (37.68–38.3) | 2273 | 25.3 (24.2–26.39) |
| **2000** | 219 | 27.38 (23.55–31.21) | 1043 | 23.36 (21.87–24.85) | 7710 | 50.66 (49.52–51.81) | 56419 | 37.74 (37.42–38.05) | 2375 | 25.07 (24.01–26.13) |
| **2001** | 219 | 26.62 (22.9–30.35) | 1171 | 24.09 (22.64–25.54) | 7768 | 49.87 (48.74–50.99) | 55109 | 36.49 (36.18–36.79) | 2523 | 25.31 (24.27–26.35) |
| **2002** | 268 | 30.99 (27.03–34.94) | 1183 | 22.91 (21.54–24.28) | 7743 | 48.83 (47.72–49.93) | 54788 | 35.86 (35.56–36.16) | 2634 | 24.79 (23.79–25.79) |
| **2003** | 226 | 26.52 (22.83–30.21) | 1231 | 22.46 (21.15–23.78) | 7710 | 47.41 (46.33–48.49) | 53564 | 34.6 (34.31–34.89) | 2736 | 24.16 (23.21–25.12) |
| **2004** | 237 | 25.71 (22.21–29.2) | 1199 | 20.97 (19.73–22.21) | 7336 | 44.34 (43.3–45.37) | 51161 | 32.68 (32.39–32.96) | 2720 | 23.09 (22.18–24.01) |
| **2005** | 258 | 26.59 (23.1–30.09) | 1275 | 20.93 (19.74–22.12) | 7623 | 44.78 (43.75–45.81) | 50280 | 31.69 (31.41–31.96) | 2863 | 23.35 (22.45–24.25) |
| **2006** | 251 | 25.43 (22.05–28.82) | 1325 | 20.57 (19.42–21.72) | 7609 | 43.49 (42.49–44.49) | 50165 | 31.15 (30.88–31.43) | 3043 | 23.33 (22.46–24.2) |
| **2007** | 259 | 24.61 (21.39–27.83) | 1390 | 20.46 (19.34–21.57) | 7639 | 42.18 (41.21–43.15) | 49768 | 30.44 (30.18–30.71) | 3116 | 22.36 (21.53–23.19) |
| **2008** | 332 | 31.39 (27.77–35.02) | 1443 | 19.95 (18.88–21.01) | 7601 | 40.83 (39.89–41.78) | 48936 | 29.5 (29.24–29.76) | 3275 | 22.18 (21.38–22.98) |
| **2009** | 320 | 28.82 (25.43–32.2) | 1447 | 18.93 (17.92–19.94) | 7555 | 39.4 (38.49–40.32) | 47421 | 28.2 (27.94–28.46) | 3390 | 21.93 (21.16–22.71) |
| **2010** | 309 | 26.62 (23.43–29.81) | 1578 | 19.67 (18.66–20.67) | 7587 | 38.32 (37.43–39.21) | 47227 | 27.73 (27.47–27.98) | 3426 | 20.99 (20.25–21.73) |
| **2011** | 345 | 28.14 (24.97–31.31) | 1619 | 18.78 (17.83–19.72) | 7655 | 37.35 (36.49–38.21) | 46441 | 26.81 (26.56–27.06) | 3586 | 20.81 (20.1–21.52) |
| **2012** | 329 | 26.05 (23.06–29.03) | 1720 | 18.93 (18.01–19.84) | 7491 | 35.29 (34.47–36.11) | 45829 | 26.01 (25.77–26.25) | 3685 | 20.19 (19.51–20.87) |
| **2013** | 373 | 28.34 (25.3–31.38) | 1673 | 16.91 (16.08–17.74) | 7539 | 34.43 (33.63–35.23) | 45745 | 25.59 (25.35–25.83) | 3901 | 20.08 (19.42–20.74) |
| **2014** | 340 | 24.47 (21.72–27.21) | 1729 | 16.49 (15.7–17.29) | 7480 | 32.62 (31.86–33.39) | 45266 | 25.01 (24.77–25.24) | 3935 | 19.13 (18.51–19.75) |
| **2015** | 362 | 25.9 (23.11–28.7) | 1893 | 17.06 (16.27–17.84) | 7747 | 32.87 (32.12–33.63) | 45479 | 24.69 (24.46–24.92) | 4130 | 18.8 (18.2–19.4) |
| **2016** | 358 | 23.39 (20.86–25.93) | 1888 | 16.2 (15.46–16.95) | 7916 | 32.49 (31.75–33.23) | 45092 | 24.18 (23.96–24.41) | 4265 | 18.51 (17.93–19.09) |
| **2017** | 402 | 25.41 (22.82–28) | 1972 | 15.99 (15.27–16.7) | 7879 | 31.41 (30.7–32.13) | 45033 | 23.83 (23.6–24.06) | 4526 | 18.66 (18.09–19.22) |
| **2018** | 373 | 23.1 (20.67–25.53) | 1983 | 15.29 (14.61–15.97) | 7699 | 29.91 (29.22–30.6) | 44950 | 23.46 (23.24–23.69) | 4723 | 18.7 (18.14–19.25) |
| **2019** | 396 | 23.55 (21.16–25.95) | 2079 | 15.41 (14.74–16.08) | 7752 | 29.34 (28.67–30.01) | 44564 | 22.96 (22.74–23.18) | 4764 | 17.95 (17.42–18.48) |
| **2020** | 417 | 24.05 (21.67–26.43) | 2234 | 15.85 (15.18–16.51) | 8140 | 29.82 (29.15–30.48) | 45052 | 23.02 (22.8–23.24) | 5153 | 18.68 (18.15–19.21) |
| **2021** | 389 | 23.97 (21.57–26.58) | 2303 | 16.71 (16.02–17.42) | 8057 | 30.27 (29.59–30.96) | 45623 | 24.1 (23.87–24.33) | 5355 | 19.25 (18.72–19.79) |
| **2022** | 384 | 22.81 (20.53–25.28) | 2408 | 16.48 (15.82–17.16) | 7930 | 28.96 (28.31–29.62) | 45903 | 23.56 (23.33–23.78) | 5504 | 18.99 (18.47–19.51) |
| **2023** | 397 | 22.91 (20.66–25.36) | 2308 | 15.21 (14.59–15.85) | 7974 | 28.5 (27.86–29.15) | 45812 | 23.53 (23.31–23.75) | 5628 | 18.65 (18.14–19.16) |
| **2024** | 449 | 24.65 (22.37–27.11) | 2366 | 14.54 (13.96–15.14) | 8043 | 27.86 (27.24–28.5) | 46333 | 23.44 (23.22–23.66) | 5852 | 18.18 (17.7–18.67) |
| **2025** | 427 | 24.08 (21.81–26.55) | 2509 | 15.45 (14.85–16.08) | 8308 | 28.88 (28.25–29.52) | 46620 | 23.57 (23.35–23.79) | 6272 | 19.43 (18.93–19.93) |
| **Total/Average** | 8839 | 25.9 (22.87–28.96) | 45892 | 18.56 (17.61–19.52) | 209148 | 37.45 (36.6–38.31) | 1304861 | 28.07 (27.81–28.32) | 105653 | 21.03 (20.31–21.76) |

*AAMR, age-adjusted mortality rate per 100,000 population; CI, confidence interval; NH, non-Hispanic.*
