# Supplementary material for: Trends and projections of colorectal carcinoma mortality in U.S. adults, 1999–2050: A CDC WONDER-based ecological time-series analysis
Source: Medicine (Baltimore). 2026 Oct 16;105(42):e51146. doi: 10.1097/MD.0000000000051146 (PMC13681118; doi:10.1097/MD.0000000000051146)
Supplement: Supplementary file 7 [file medi-105-e51146-s007.docx]

**Trends and Projections of Colorectal Carcinoma Mortality in U.S. Adults, 1999–2050: A CDC WONDER-Based Ecological Time-Series Analysis**

**Supplemental Table 6:** Trends in Age-Adjusted Mortality Rates and Deaths from Colorectal cancer among United States Adults Aged 25 and Older stratified by Urbanization, 1999–2020.

| **Year** | **Metropolitan** | | **Non-metropolitan** | |
| --- | --- | --- | --- | --- |
|  | **Deaths** | **AAMR (95% CI)** | **Deaths** | **AAMR (95% CI)** |
| **1999** | 54163 | 37.83 (37.51–38.15) | 13171 | 39.6 (38.92–40.28) |
| **2000** | 54526 | 37.58 (37.26–37.89) | 13240 | 39.4 (38.73–40.08) |
| **2001** | 53731 | 36.33 (36.02–36.64) | 13059 | 38.6 (37.93–39.26) |
| **2002** | 53466 | 35.51 (35.21–35.81) | 13150 | 38.51 (37.85–39.17) |
| **2003** | 52776 | 34.42 (34.13–34.71) | 12691 | 36.74 (36.09–37.38) |
| **2004** | 50348 | 32.28 (31.99–32.56) | 12305 | 35.23 (34.61–35.85) |
| **2005** | 50077 | 31.44 (31.16–31.72) | 12222 | 34.61 (34–35.23) |
| **2006** | 50333 | 30.92 (30.65–31.19) | 12060 | 33.58 (32.98–34.18) |
| **2007** | 49835 | 29.95 (29.68–30.21) | 12337 | 33.86 (33.25–34.46) |
| **2008** | 49398 | 29.06 (28.8–29.31) | 12189 | 33.03 (32.44–33.62) |
| **2009** | 48420 | 27.83 (27.59–28.08) | 11713 | 31.34 (30.77–31.92) |
| **2010** | 48155 | 27.17 (26.93–27.42) | 11972 | 31.69 (31.12–32.26) |
| **2011** | 47890 | 26.23 (25.99–26.47) | 11756 | 30.74 (30.18–31.3) |
| **2012** | 47406 | 25.34 (25.11–25.57) | 11648 | 29.93 (29.37–30.48) |
| **2013** | 47568 | 24.84 (24.62–25.07) | 11663 | 29.64 (29.09–30.19) |
| **2014** | 47396 | 24.11 (23.89–24.33) | 11354 | 28.57 (28.03–29.11) |
| **2015** | 47888 | 23.72 (23.51–23.94) | 11723 | 28.98 (28.44–29.52) |
| **2016** | 48067 | 23.34 (23.12–23.55) | 11452 | 28 (27.47–28.52) |
| **2017** | 48178 | 22.87 (22.66–23.08) | 11634 | 28.12 (27.59–28.64) |
| **2018** | 48032 | 22.34 (22.14–22.54) | 11696 | 27.91 (27.39–28.43) |
| **2019** | 47827 | 21.77 (21.57–21.97) | 11728 | 27.57 (27.05–28.08) |
| **2020** | 49110 | 21.97 (21.77–22.16) | 11886 | 27.77 (27.26–28.29) |
| **Total/Average** | 1094590 | 28.49 (28.24–28.74) | 266649 | 32.43 (31.84–33.01) |

*AAMR, age-adjusted mortality rate per 100,000 population; CI, confidence interval.*
