# Supplementary material for: Trends and projections of colorectal carcinoma mortality in U.S. adults, 1999–2050: A CDC WONDER-based ecological time-series analysis
Source: Medicine (Baltimore). 2026 Oct 16;105(42):e51146. doi: 10.1097/MD.0000000000051146 (PMC13681118; doi:10.1097/MD.0000000000051146)
Supplement: Supplementary file 8 [file medi-105-e51146-s008.docx]

**Trends and Projections of Colorectal Carcinoma Mortality in U.S. Adults, 1999–2050: A CDC WONDER-Based Ecological Time-Series Analysis**

**Supplemental Table 7:** Trends in Age-Adjusted Mortality Rates and Deaths from Colorectal cancer among United States Adults Aged 25 and Older stratified by Census region, 1999–2025.

| **Year** | **Northeast** | | **Midwest** | | **South** | | **West** | |
| --- | --- | --- | --- | --- | --- | --- | --- | --- |
|  | **Deaths** | **AAMR (95% CI)** | **Deaths** | **AAMR (95% CI)** | **Deaths** | **AAMR (95% CI)** | **Deaths** | **AAMR (95% CI)** |
| **1999** | 15559 | 41.74 (41.09–42.4) | 17205 | 40.85 (40.24–41.46) | 22747 | 36.58 (36.1–37.05) | 11823 | 33.69 (33.08–34.3) |
| **2000** | 15474 | 41.04 (40.4–41.69) | 16892 | 39.8 (39.2–40.4) | 23403 | 37.14 (36.67–37.62) | 11997 | 33.58 (32.98–34.18) |
| **2001** | 14979 | 39.34 (38.71–39.98) | 16688 | 38.84 (38.25–39.43) | 23044 | 35.89 (35.42–36.35) | 12079 | 32.9 (32.32–33.49) |
| **2002** | 14862 | 38.55 (37.93–39.17) | 16478 | 37.91 (37.33–38.49) | 23311 | 35.66 (35.2–36.12) | 11965 | 31.81 (31.24–32.39) |
| **2003** | 14305 | 36.66 (36.06–37.26) | 16244 | 36.9 (36.33–37.47) | 22683 | 34.07 (33.62–34.51) | 12235 | 31.78 (31.21–32.34) |
| **2004** | 13559 | 34.38 (33.8–34.96) | 15356 | 34.44 (33.9–34.99) | 22070 | 32.53 (32.1–32.96) | 11668 | 29.65 (29.11–30.19) |
| **2005** | 13118 | 32.93 (32.37–33.5) | 15212 | 33.67 (33.14–34.21) | 22106 | 31.85 (31.43–32.27) | 11863 | 29.31 (28.78–29.83) |
| **2006** | 12927 | 32.13 (31.58–32.69) | 15302 | 33.32 (32.79–33.85) | 22224 | 31.2 (30.79–31.61) | 11940 | 28.76 (28.24–29.28) |
| **2007** | 12747 | 31.27 (30.72–31.81) | 15137 | 32.46 (31.95–32.98) | 22442 | 30.71 (30.31–31.11) | 11846 | 27.79 (27.29–28.29) |
| **2008** | 12528 | 30.33 (29.8–30.87) | 14967 | 31.67 (31.16–32.18) | 22350 | 29.86 (29.47–30.26) | 11742 | 26.78 (26.29–27.26) |
| **2009** | 12159 | 29.04 (28.52–29.56) | 14313 | 29.77 (29.28–30.26) | 21957 | 28.63 (28.25–29.01) | 11704 | 25.96 (25.49–26.44) |
| **2010** | 12058 | 28.37 (27.85–28.88) | 14345 | 29.53 (29.05–30.02) | 21934 | 28.06 (27.68–28.43) | 11790 | 25.6 (25.14–26.07) |
| **2011** | 11520 | 26.71 (26.22–27.21) | 14151 | 28.59 (28.11–29.07) | 21944 | 27.17 (26.81–27.53) | 12031 | 25.19 (24.74–25.65) |
| **2012** | 11430 | 26.07 (25.59–26.56) | 13796 | 27.33 (26.87–27.79) | 22014 | 26.54 (26.19–26.9) | 11814 | 24.04 (23.6–24.47) |
| **2013** | 11228 | 25.23 (24.76–25.7) | 13943 | 27.23 (26.77–27.68) | 22225 | 26.11 (25.76–26.46) | 11835 | 23.42 (22.99–23.84) |
| **2014** | 10988 | 24.27 (23.81–24.73) | 13672 | 26.31 (25.86–26.76) | 22280 | 25.5 (25.16–25.84) | 11810 | 22.68 (22.27–23.1) |
| **2015** | 10974 | 23.97 (23.51–24.43) | 13699 | 25.87 (25.43–26.31) | 22777 | 25.35 (25.02–25.68) | 12161 | 22.63 (22.22–23.03) |
| **2016** | 10721 | 23.24 (22.79–23.69) | 13590 | 25.26 (24.83–25.69) | 23053 | 25 (24.67–25.33) | 12155 | 22.12 (21.72–22.52) |
| **2017** | 10670 | 22.56 (22.12–22.99) | 13505 | 24.85 (24.42–25.28) | 23281 | 24.66 (24.34–24.99) | 12356 | 22.03 (21.63–22.42) |
| **2018** | 10330 | 21.7 (21.27–22.13) | 13672 | 24.69 (24.27–25.12) | 23334 | 24.13 (23.82–24.45) | 12392 | 21.45 (21.07–21.84) |
| **2019** | 10167 | 21.02 (20.6–21.43) | 13397 | 23.82 (23.41–24.23) | 23576 | 23.86 (23.55–24.17) | 12415 | 21.08 (20.7–21.46) |
| **2020** | 10547 | 21.58 (21.16–22) | 13628 | 24.08 (23.67–24.5) | 24092 | 23.84 (23.53–24.14) | 12729 | 21.21 (20.84–21.59) |
| **2021** | 10159 | 21.09 (20.67–21.51) | 13705 | 25.01 (24.58–25.44) | 24855 | 25.21 (24.89–25.53) | 13008 | 22.55 (22.15–22.94) |
| **2022** | 10084 | 20.32 (19.92–20.73) | 13652 | 24.06 (23.65–24.47) | 25231 | 24.74 (24.43–25.05) | 13162 | 21.97 (21.59–22.35) |
| **2023** | 10105 | 20.39 (19.99–20.8) | 13485 | 23.71 (23.3–24.12) | 25580 | 24.78 (24.47–25.09) | 12949 | 21.4 (21.03–21.78) |
| **2024** | 10203 | 20.16 (19.76–20.57) | 13813 | 23.8 (23.39–24.21) | 25741 | 24.26 (23.96–24.56) | 13286 | 21.42 (21.05–21.8) |
| **2025** | 10233 | 20.1 (19.7–20.5) | 14131 | 24.34 (23.93–24.75) | 26186 | 24.66 (24.35–24.96) | 13586 | 21.96 (21.59–22.34) |
| **Total/Average** | 323634 | 27.93 (27.43–28.44) | 393978 | 29.56 (29.08–30.04) | 626440 | 28.44 (28.07–28.81) | 330341 | 25.66 (25.2–26.12) |

*AAMR, age-adjusted mortality rate per 100,000 population; CI, confidence interval.*
