# Supplementary material for: Trends and projections of colorectal carcinoma mortality in U.S. adults, 1999–2050: A CDC WONDER-based ecological time-series analysis
Source: Medicine (Baltimore). 2026 Oct 16;105(42):e51146. doi: 10.1097/MD.0000000000051146 (PMC13681118; doi:10.1097/MD.0000000000051146)
Supplement: Supplementary file 9 [file medi-105-e51146-s009.docx]

**Trends and Projections of Colorectal Carcinoma Mortality in U.S. Adults, 1999–2050: A CDC WONDER-Based Ecological Time-Series Analysis**

**Supplemental Table 8:** Trends in Age-Adjusted Mortality Rates and Deaths from Colorectal cancer among United States Adults Aged 25 and Older stratified by State, 1999–2025.

| **State** | **1999-2020** | | **2021-2025** | | **1999-2025** | **Weighted Mean AAMR 1999-2025** |
| --- | --- | --- | --- | --- | --- | --- |
|  | **Deaths** | **AAMR (95% CI)** | **Deaths** | **AAMR (95% CI)** | **Deaths** |  |
| **Alabama** | 22291 | 29.59 (29.2–29.98) | 5439 | 25.99 (25.29–26.71) | 27730 | 28.923 |
| **Alaska** | 1985 | 28.68 (27.32–30.03) | 566 | 24.76 (22.67–27.01) | 2551 | 27.954 |
| **Arizona** | 23848 | 23.88 (23.57–24.18) | 6793 | 21.56 (21.03–22.09) | 30641 | 23.45 |
| **Arkansas** | 15045 | 31.84 (31.33–32.36) | 3602 | 29.11 (28.14–30.1) | 18647 | 31.334 |
| **California** | 135944 | 26.16 (26.02–26.3) | 31693 | 21.46 (21.22–21.7) | 167637 | 25.29 |
| **Colorado** | 16756 | 24.97 (24.59–25.36) | 4534 | 21.45 (20.82–22.1) | 21290 | 24.318 |
| **Connecticut** | 15791 | 25.58 (25.18–25.99) | 2822 | 17.66 (17–18.35) | 18613 | 24.113 |
| **Delaware** | 4054 | 27.47 (26.62–28.32) | 1075 | 23.11 (21.7–24.61) | 5129 | 26.663 |
| **District of Columbia** | 2880 | 33.7 (32.46–34.94) | 513 | 23.56 (21.55–25.72) | 3393 | 31.822 |
| **Florida** | 93054 | 25.29 (25.13–25.46) | 23403 | 21.25 (20.97–21.53) | 116457 | 24.542 |
| **Georgia** | 34783 | 27.67 (27.38–27.97) | 9872 | 24.74 (24.24–25.24) | 44655 | 27.127 |
| **Hawaii** | 5642 | 24.52 (23.87–25.16) | 1113 | 18.74 (17.59–19.96) | 6755 | 23.45 |
| **Idaho** | 5617 | 24.8 (24.15–25.46) | 1708 | 23.21 (22.1–24.37) | 7325 | 24.506 |
| **Illinois** | 60020 | 30.89 (30.64–31.14) | 11800 | 22.91 (22.49–23.34) | 71820 | 29.412 |
| **Indiana** | 31287 | 31.42 (31.07–31.77) | 7282 | 26.88 (26.25–27.52) | 38569 | 30.579 |
| **Iowa** | 17037 | 31.21 (30.73–31.68) | 3372 | 24.87 (24.02–25.75) | 20409 | 30.036 |
| **Kansas** | 13068 | 28.76 (28.26–29.26) | 2857 | 24.5 (23.59–25.43) | 15925 | 27.971 |
| **Kentucky** | 22514 | 33.62 (33.18–34.07) | 5402 | 29.6 (28.8–30.42) | 27916 | 32.876 |
| **Louisiana** | 21506 | 32.03 (31.6–32.46) | 4816 | 26.76 (25.99–27.55) | 26322 | 31.054 |
| **Maine** | 7013 | 28.45 (27.78–29.13) | 1440 | 21.21 (20.09–22.39) | 8453 | 27.109 |
| **Maryland** | 25817 | 30.09 (29.72–30.46) | 5870 | 23.49 (22.88–24.11) | 31687 | 28.868 |
| **Massachusetts** | 29856 | 26.92 (26.61–27.23) | 5198 | 17.57 (17.09–18.07) | 35054 | 25.189 |
| **Michigan** | 46272 | 28.78 (28.51–29.04) | 9905 | 23.27 (22.8–23.74) | 56177 | 27.76 |
| **Minnesota** | 22275 | 26.55 (26.2–26.9) | 5178 | 22.16 (21.55–22.79) | 27453 | 25.737 |
| **Mississippi** | 15683 | 35.35 (34.79–35.9) | 3925 | 33.11 (32.06–34.19) | 19608 | 34.935 |
| **Missouri** | 29620 | 30.36 (30.01–30.71) | 6671 | 25.89 (25.26–26.53) | 36291 | 29.532 |
| **Montana** | 4452 | 25.97 (25.2–26.74) | 1160 | 23.84 (22.45–25.31) | 5612 | 25.576 |
| **Nebraska** | 9832 | 32.85 (32.19–33.5) | 2128 | 27.41 (26.24–28.63) | 11960 | 31.843 |
| **Nevada** | 10749 | 29.02 (28.46–29.58) | 3060 | 24.71 (23.83–25.63) | 13809 | 28.222 |
| **New Hampshire** | 5842 | 27.09 (26.39–27.8) | 1258 | 19.58 (18.48–20.74) | 7100 | 25.699 |
| **New Jersey** | 43325 | 30.27 (29.98–30.55) | 8068 | 20.68 (20.23–21.15) | 51393 | 28.494 |
| **New Mexico** | 7912 | 25.54 (24.97–26.1) | 2137 | 23.83 (22.8–24.91) | 10049 | 25.223 |
| **New York** | 89289 | 28.35 (28.16–28.54) | 16399 | 19.49 (19.19–19.8) | 105688 | 26.709 |
| **North Carolina** | 38935 | 27.17 (26.9–27.44) | 9894 | 22.91 (22.45–23.38) | 48829 | 26.381 |
| **North Dakota** | 3609 | 30.07 (29.07–31.06) | 698 | 22.49 (20.81–24.28) | 4307 | 28.666 |
| **Ohio** | 62296 | 32.55 (32.3–32.81) | 12395 | 25.02 (24.57–25.47) | 74691 | 31.156 |
| **Oklahoma** | 18744 | 32.24 (31.77–32.7) | 4743 | 31.45 (30.55–32.38) | 23487 | 32.094 |
| **Oregon** | 17162 | 27.14 (26.73–27.54) | 4164 | 23.45 (22.73–24.19) | 21326 | 26.457 |
| **Pennsylvania** | 72926 | 31.39 (31.16–31.62) | 13943 | 23.75 (23.34–24.16) | 86869 | 29.975 |
| **Rhode Island** | 5581 | 29.61 (28.82–30.39) | 950 | 19.51 (18.26–20.84) | 6531 | 27.74 |
| **South Carolina** | 20772 | 28.72 (28.32–29.12) | 5451 | 24.14 (23.48–24.81) | 26223 | 27.872 |
| **South Dakota** | 4404 | 31.07 (30.15–32) | 996 | 26.82 (25.13–28.61) | 5400 | 30.283 |
| **Tennessee** | 30278 | 30.91 (30.56–31.26) | 7649 | 27.02 (26.4–27.65) | 37927 | 30.19 |
| **Texas** | 88664 | 27.93 (27.75–28.12) | 25413 | 25.39 (25.07–25.71) | 114077 | 27.46 |
| **Utah** | 6288 | 20.99 (20.47–21.52) | 1808 | 18.73 (17.86–19.63) | 8096 | 20.571 |
| **Vermont** | 3227 | 30 (28.96–31.05) | 706 | 23.28 (21.53–25.16) | 3933 | 28.756 |
| **Virginia** | 31812 | 27.33 (27.03–27.63) | 7934 | 23.1 (22.59–23.62) | 39746 | 26.547 |
| **Washington** | 25685 | 25.62 (25.31–25.94) | 6618 | 22.35 (21.8–22.9) | 32303 | 25.014 |
| **West Virginia** | 12015 | 35.82 (35.17–36.46) | 2592 | 31.34 (30.1–32.62) | 14607 | 34.99 |
| **Wisconsin** | 25472 | 27.19 (26.85–27.52) | 5504 | 22.01 (21.42–22.62) | 30976 | 26.231 |
| **Wyoming** | 2310 | 27.81 (26.67–28.96) | 637 | 26.67 (24.59–28.91) | 2947 | 27.599 |

*AAMR, age-adjusted mortality rate per 100,000 population; CI, confidence interval.*
